# Vision SLAM algorithm for wheeled robots integrating multiple sensors

**DOI:** 10.1371/journal.pone.0301189

**Published:** 2024-03-28

**Authors:** Weihua Zhou, Rougang Zhou

**Affiliations:** 1 School of Computer and Information Technology (School of Big Data), Shanxi University, Taiyuan, 030002, China; 2 School of Mechanical Engineering, Hangzhou Dianzi University, Hangzhou, 310018, China; Government College University Lahore, PAKISTAN

## Abstract

Wheeled robots play a crucial role in driving the autonomy and intelligence of robotics. However, they often encounter challenges such as tracking loss and poor real-time performance in low-texture environments. In response to these issues, this research proposes a real-time localization and mapping algorithm based on the fusion of multiple features, utilizing point, line, surface, and matrix decomposition characteristics. Building upon this foundation, the algorithm integrates multiple sensors to design a vision-based real-time localization and mapping algorithm for wheeled robots. The study concludes with experimental validation on a two-wheeled robot platform. The results indicated that the multi-feature fusion algorithm achieved the highest average accuracy in both conventional indoor datasets (84.57%) and sparse-feature indoor datasets (82.37%). In indoor scenarios, the vision-based algorithm integrating multiple sensors achieved an average accuracy of 85.4% with a processing time of 64.4 ms. In outdoor scenarios, the proposed algorithm exhibited a 14.51% accuracy improvement over a vision-based algorithm without closed-loop detection. In summary, the proposed method demonstrated outstanding accuracy and real-time performance, exhibiting favorable application effects across various practical scenarios.

## 1. Introduction

The role of artificial intelligence in the robotics industry is becoming increasingly significant, empowering robots by improving their self-learning abilities and applying them in multiple scenarios. Deep learning, computer vision, and other technologies in artificial intelligence can enable robots to better understand and execute tasks, improving production efficiency. In the field of mobile robots, AI not only improves production efficiency, but also ensures functional safety and information security, which has spawned new applications such as autonomous vehicle, cooperative robots and unmanned aerial vehicles [1,2]. According to statistics, the Chinese mobile robot market has been in a rapid development stage in recent years, driven by manufacturing automation, service industry upgrading, and technological innovation. And the size of China’s mobile robot market is showing an upward trend year by year. In 2022, the market size of China’s mobile robot industry is about 8.902 billion yuan, mainly concentrated in the East China region, accounting for 30.11%. In addition, the production of China’s mobile robot industry in 2022 is about 44900 units, and the demand is about 47100 units. Therefore, the application and development prospects of mobile robots are broad [3]. These robots find applications in industrial production, logistics, and other fields. Key technologies influencing wheeled robots include perception and planning, localization, and control [4]. However, the accuracy of wheeled robot localization in complex unknown environments remains a challenge [5,6]. Commonly used methods for robot localization include Simultaneous Localization and Mapping (SLAM), which leverage sensor data to construct maps and determine robot positions [7]. However, SLAM algorithms often face accuracy and tracking loss issues in low-texture environments. To address these challenges, the research introduces an SLAM optimization algorithm based on multi-feature fusion (OMFF-SLAM). Combining this with an Inertial Measurement Unit (IMU), multiple sensors, and the Visual-Inertial Fusion Positioning Optimization algorithm (VIFPO), the study proposes a vision SLAM algorithm for wheeled robots integrating multiple sensors (IMS-VSLAM). In the final phase, a Two Wheeled Robot (TWR) platform was constructed to evaluate the performance of the algorithms mentioned above. The research aimed to acquire more accurate environmental information to assist wheeled robots in better understanding their surroundings. This, in turn, enhances the precision of localization and navigation, enabling real-time navigation in complex environments. The objective is to enable robots to respond more swiftly and accurately to changes in their surroundings in various practical applications. The innovation in the research primarily revolves around two points. Firstly, the introduction of the OMFF-SLAM algorithm addresses the tracking loss issue encountered by classical SLAM algorithms in low-texture scenarios. Secondly, the design of IMS-VSLAM contributes to improving the localization accuracy of wheeled robots. The research structure is divided into four main parts. The first part involves a review of relevant research outcomes. The second part entails the design of the OMFF-SLAM and IMS-VSLAM algorithms, along with the construction of the TWR robot platform. The third part validates the effectiveness of the proposed methods. The final part concludes the research. The contribution of the research lies in proposing the OMFF-SLAM algorithm and IMS-VSLAM algorithm to solve the problems of poor tracking performance in texture scenes, decreased accuracy and tracking loss in more challenging scenes using traditional methods. It also uses IMU and encoder to visually track the motion trajectory when lost, to compensate for the shortcomings of the phase machine and provide a new method for wheeled robots with higher accuracy and real-time requirements. The aim of this paper is to provide new avenues for the hybrid utilization of electric vehicles, renewable energy systems and artificial intelligence through the proposed algorithm, which can help reduce unnecessarily high pollution in this field.

## 2. Related works

With the continuous development of artificial intelligence, intelligent robots have emerged as crucial entities in today’s society. They are important in various fields and positions, necessitating continuous research as the relevant technologies are yet to mature. Wheeled robots, utilizing wheels as their mobile units, offer high mobility and adaptability in diverse environments. M. Xiong et al. proposed a hierarchical semantic fusion strategy for depth estimation under various weather conditions, especially under extreme conditions, and determined the adaptive contribution of each sensor and weighted sensor data for features. The proposed method was compared with the limit model and extended model, and the results showed that the proposed method had robustness and excellent performance. In addition, the performance of cameras and radars in foggy environments was studied through visually generated masks. The results showed that the importance of radar sensors significantly increased under extreme weather conditions [8]. H. Zhou et al. proposed a hybrid obstacle avoidance method combining a three-dimensional (3D) target detection approach with a model predictive controller to enhance the obstacle avoidance performance of wheeled mobile robots. The results indicated that this method met the application requirements of wheeled robots in unstructured environments. Additionally, the study optimized the model predictive controller by incorporating the distance between obstacles and robots, ensuring a safe distance [9]. A. Alhalabi et al. introduced a drift compensation technology for a wheeled robot platform based on multi-sensor fusion. This technique combined information from encoders and acceleration sensors to estimate drift in the acceleration dimension. Experimental results demonstrated the effectiveness of this method in reducing the localization drift of wheeled robots [10]. T. Huang et al. found that visual semantic fusion is a key technology for understanding autonomous driving scenes, and its accuracy is affected by changes in lighting. Therefore, they studied and designed an emerging multi-exposure semantic fusion method to enhance the visual semantics of autonomous driving. Experimental results showed that this method could effectively recover lost features in lighting change images and improve the accuracy of subsequent semantic fusion [11].

In today’s rapidly advancing technological landscape, the field of visual SLAM has experienced remarkable development alongside the continuous evolution of intelligent robots. When an autonomous mobile robot operates within a given environment, visual SLAM assesses its position in relation to the surroundings using information obtained from cameras. During the robot’s movement, it continuously updates and constructs maps, plans paths, and performs self-localization based on the perceived data from cameras and estimated positions. S. Wen et al. aimed to enhance obstacle recognition speed using monocular depth perception. They proposed a path planning algorithm optimized by a probabilistic duel deep reinforcement learning algorithm, integrating it with visual SLAM for autonomous navigation and environmental mapping. The results demonstrated successful autonomous navigation in indoor environments, encompassing various static and dynamic obstacles [12]. J. Cremona et al. developed several SLAM systems due to the highly repetitive scenes and visual difficulties caused by wind induced crop leaf movement when mobile autonomous robots perform tasks such as sowing, harvesting, and weed control. The study evaluated the use of wheeled robots on sensor datasets recorded in soybeans, and the results showed that ORB-SLAM3 and S-MSCKF systems could perform better with reliability in agricultural applications [13]. Z. H. Wang et al. artificially confirmed that heterogeneous robots have the potential to perform more complex tasks through collaboration, and demanded that the vehicle navigation system can work safely and effectively in areas with weak or even unreachable GNSS signals. They proposed a collaborative system based on the PRB-SLAM2 system to adapt to actual environmental exploration and perception. Indoor flight experiments verified the effectiveness of the proposed system [14]. Recognizing the challenge of executing tasks in dynamic environments, A. Li et al. introduced a novel sparse-feature-based visual SLAM. Through experiments with various datasets, they demonstrated improved performance in various complex scenarios [15].

In summary, research outcomes in the intersection of wheeled robots and visual SLAM technology abound. However, existing real-time visual SLAM technologies for robots exhibit shortcomings, especially in addressing wheel slippage errors. To tackle this, the study introduces the OMFF-SLAM and IMS-VSLAM algorithms and establishes the TWR robot platform.

## 3. Design of IMS-VSLAM algorithm for wheeled robots

As robotics technology continues to advance, the exploration of mapping and self-localization in unknown environments for robots has gained significant attention. Visual SLAM algorithms play a crucial role in addressing these challenges. Leveraging machine vision and image processing, vision SLAM enables robots to perceive their surroundings in unknown environments, facilitating map construction and self-localization [16–18]. This study addresses the tracking loss issue in traditional SLAM algorithms by introducing the OMFF-SLAM algorithm. Subsequently, this algorithm is integrated with multiple sensors, IMU, and the VIFPO algorithm to form the IMS-VSLAM algorithm. The performance of the algorithm is validated using the TWR robot platform.

### 3.1. Construction of OMFF-SLAM algorithm

In order to facilitate subsequent understanding, the study summarized the English abbreviations in the article and obtained Table 1.

**Table 1:** List of English abbreviations.

Abbreviation	Full Name
SLAM	Simultaneous Localization and Mapping
OMFF-SLAM	SLAM Optimization Based on Multi Feature Fusion
IMU	Inertial Measurement Unit
VIFPO	Visual Inner Fusion Positioning Optimization
IMS-VSLAM	Visual SLAM for Wheeled Robots Integrating Multiple Sensors
TWR	Two Wheeled Robot
ORB	ORiented Brief
MF	Matrix Factorization
NRO	Nonlinear Optimization
MMWH	Mixed Manhattan World Hypothesis
LMO	Local Map Optimization
FPODAPT	Feature Points Obtained from Directional Acceleration Phase Testing
RI-BRIEF	Rotational Invariance
OpenCV	Open Source Computer Vision Library
FLD	Fisher Linear discrimination
LBD	Line Band Discriptor
AHC	Aggregated Hierarchical Clustering
LM	Levinberg Marquardt
V-IMU-E	Vision IMU Encoder
KLTOF	Kanade Lucas Tomasi Optical Flow
ER	Encoder
OS	Operating System
ROS	Robot Operating System
LiDARO-SLAM	Improved SLAM Based on LiDAR
BWFIN-SLAM	SLAM Based on the Fusion of Binocular Wire Features and Inertial Navigation
RMSE	Root Mmean Square Error
AT	Average Time
RI	Regular Indoor
SIF	Sparse Indoor Features
WCLD-IMS-VSLAM	IMS-VSLAM with Closed-loop Detection
WOCLD-IMS-VSLAM	IMS-VSLAM without Closed-loop Detection
OD	Outdoor Dataset

**Fig 1:**
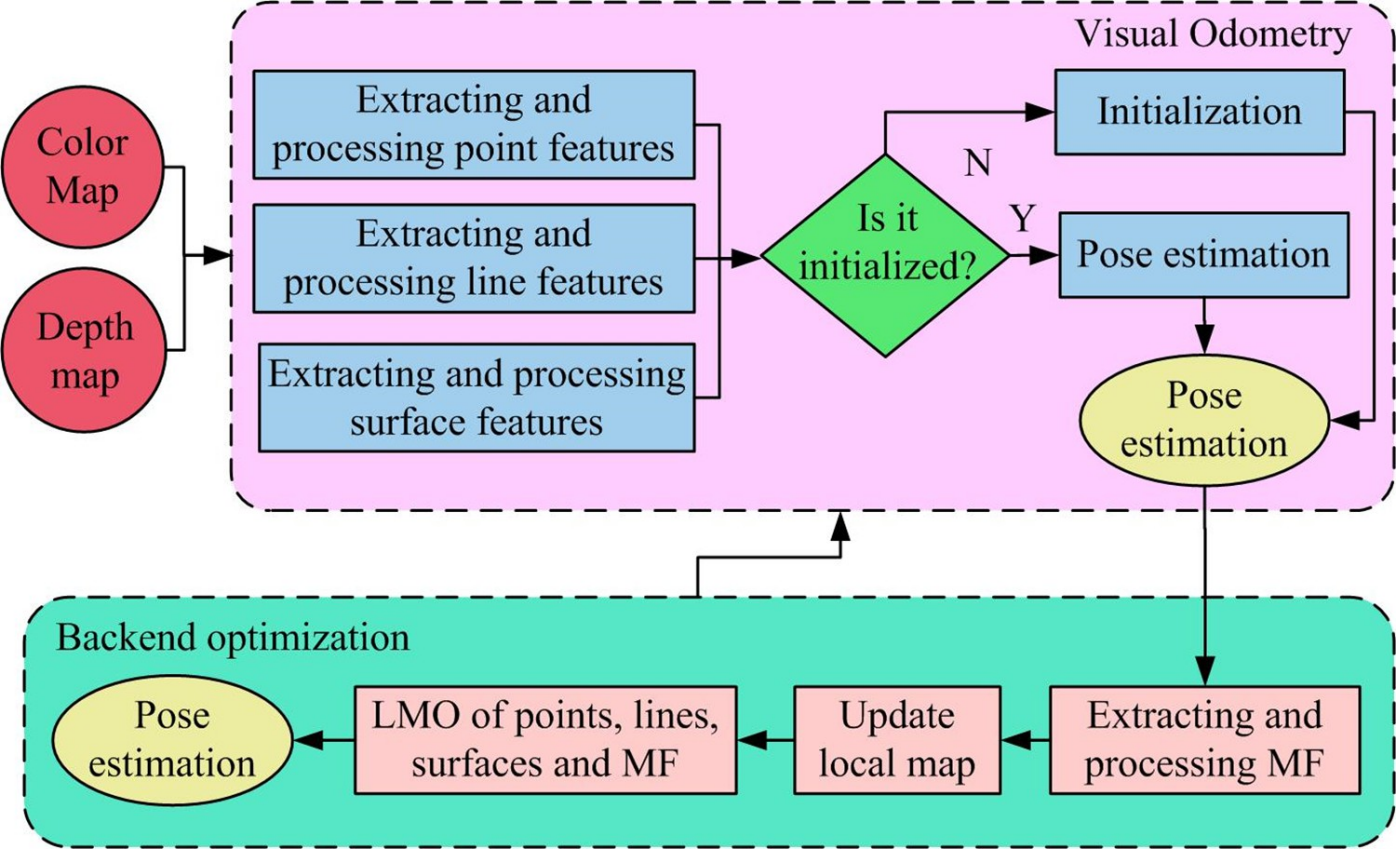
OMFF-SLAM algorithm process.

Due to the tracking loss problem in traditional SLAM algorithms, this research enhances the ORiented Brief (ORB) SLAM2 by incorporating three types of features: lines, surfaces, and Matrix Factorization (MF). Nonlinear Optimization (NRO) is employed to minimize the constructed objective function. The application effectiveness is further enhanced through the integration of Mixed Manhattan World Hypothesis (MMWH) and Local Map Optimization (LMO) [19–21]. The workflow of the OMFF-SLAM algorithm is illustrated in Fig 1.

In Fig 1, it mainly includes two parts: visual odometry and backend optimization. The visual odometry is responsible for real-time processing of color and dark images, completing initialization and estimation of the current frame pose, and deciding whether to insert the current frame as a keyframe into the backend. Firstly, it is necessary to extract and process the point, line, and surface features in the current frame. If initialization is not completed, the initial map is constructed through initialization. Otherwise, it is necessary to establish the matching relationship between the point, line, and surface features in the current frame and map points, lines, and surfaces through matching. NRO methods are used to fuse point, line, and surface features to estimate the current pose. Finally, based on the tracking quality and backend optimization space, it is needed to decide whether to insert keyframes into the backend. In the backend optimization part. The research is mainly responsible for keyframes, reconstructing points, lines, and surfaces of the new map through triangulation, and estimating state variables such as keyframe points, lines, and surfaces in the local map through nonlinear optimization methods. In the feature extraction section of the visual odometry in the OMFF-SLAM algorithm, ORB point features include Feature Points Obtained from Directional Acceleration Phase Testing (FPODAPT) and Rotational Invariance-Binary Robust Independent Elementary Feature (RI-BRIEF) with rotational invariance. The key steps for extracting FPODAPT keypoints are as follows. The current pixel point and luminance are designated as *p* and *l*, respectively. A circle with a radius of 3, centered at *p*, containing 16 pixel points is selected. Subsequently, it is determined whether *p* is an FPODAPT keypoint. If so, the surrounding image block *A* is selected, and $\vec{pc}$ is taken as the direction, with *c* as the centroid, calculated as shown in Eq (1).


$$\left\{ \begin{array}{l} c=(\frac{m_{10}}{m_{00}},\frac{m_{01}}{m_{00}})\\ m_{ad}=\sum_{x,y\in A}x^{a}y^{d}I(x,y) \end{array}\right.$$ (1)

In Eq (1), *m*_*ad*_ and *I*(*x*,*y*) represent the quality and pixel value of each pixel, respectively. The extraction process of RI-BRIEF is as follows. First, $\vec{pc}$ is used as the reference direction, then an invariant set of 256 pairs of pixels is selected. These pixels are then paired with binary-described points. The Hamming distance between the binary RI-BRIEF of the two points is calculated. If this value is less than the threshold, the point features are considered a match, with the threshold range set between [50,100]. Line features include Fisher Linear Discrimination (FLD) keypoints and binary Line Band Descriptor (LBD) descriptors provided by the Open Source Computer Vision Library (OpenCV). The extraction process of the binary LBD descriptor is as follows. First, relevant information about the line segment is obtained through FLD key points. Then, the key points are considered as the midline. An area with *m* stripes is selected, each with a width of *w* pixels. The midpoint of the key line is set as the origin, with the horizontal and vertical directions of the coordinate axis defined as *d*_*H*_ and *d*_*V*_, respectively. The gradient of each pixel in these two directions is defined, and the gradients are divided based on whether they are greater than or less than 0. A 4-dimensional vector is eventually obtained through accumulation. Each region’s *n* pixel stripes form the matrix *B*_*i*_. The mean vector *μ*_*i*_ and the standard deviation vector *σ*_*i*_ are computed for each row of *B*_*i*_, forming a floating-point vector, as shown in Eq (2).


$$LBD={\left(B_{1}^{T},B_{2}^{T},\cdots ,B_{m}^{T}\right)}^{T}$$ (2)

Finally, it is converted into a binary vector. 32 pairs of vectors are selected and a pairwise comparison is performed on each dimension to obtain the binary LBD descriptor. Surface features are extracted through Aggregated Hierarchical Clustering (AHC), and MF features are extracted through both lines and surfaces. When initiating the OMFF-SLAM algorithm, initialization is required to establish the initial map. Once initialized, the algorithm proceeds to pose estimation. Using NRO, the current frame’s camera coordinate system *c*_*k*_ C is estimated based on the current frame’s pose $\left(R_{c_{k}}^{w},p_{c_{k}}^{w}\right)$, solving according to the inverse $\left(R_{w}^{c_{k}},p_{w}^{c_{k}}\right)$ [22]. NRO is widely used in solving least squares problems, as shown in Eq (3).


$$x'=\underset{x}{\arg\;\min}\frac{1}{2}{\left\|f(x)\right\|}^{2}$$ (3)

Eq (3) represents a multidimensional state vector, where *x* denotes the state vector, *x*’ represents the optimal solution corresponding to *x*, and *f*(*x*) is an arbitrary nonlinear function. In solving the least squares problem, the OMFF-SLAM algorithm utilizes the Levenberg-Marquardt (LM) method and integrates frame-to-frame optimization, relocalization, and LMO estimation $\left(R_{c_{k}}^{w},p_{c_{k}}^{w}\right)$ as described in references. The objective function established is shown in Eq (4).


$$F\left(R_{c_{k}}^{w},p_{c_{k}}^{w}\right)={\sum_{h=0}^{H_{I}}\left\|e_{p,k-h}\right\|}_{\Sigma_{p,k-h}}^{2}+{\sum_{g=0}^{G_{I}}\left\|e_{l,k-g}\right\|}_{\Sigma_{l,k-g}}^{2}+{\sum_{j=0}^{J_{I}}\left\|e_{\pi ,k-j}\right\|}_{\Sigma_{\pi ,k-j}}^{2}$$ (4)

In Eq (4), Σ_*p*,*k−h*_, Σ_*l*,*k−g*_, Σ_*π*,*k−j*_, *e*_*p*,*k−h*_, *e*_*l*,*k−g*_, and *e*_*π*,*k−j*_ correspond to the measurement noise covariance matrices and measurement error terms for points, lines, and surfaces, respectively. *H*_1_, *G*_1_, and *J*_1_ represent the total number of corresponding points, lines, and surfaces in the matching set. Additionally, by using the Jacobian matrices *e*_*p*,*k−h*_, *e*_*l*,*k−g*_, and *e*_*π*,*k−j*_, the g2o library can be invoked to automatically solve the Jacobian matrices for all error terms. To update $\left(R_{c_{k}}^{w},p_{c_{k}}^{w}\right)$ through cumulative summation, $R_{w}^{c_{k}}$ is transformed into a non-redundant Lie algebra $\phi_{w}^{c_{k}}$ using the rotation matrix. If the tracking task of the second newest frame is successful, the relocation step can be ignored; otherwise, the current frame needs to be relocalized, and $\left(R_{c_{k}}^{w},p_{c_{k}}^{w}\right)$ is estimated. In the LMO method, the objective function is established through frame-to-frame optimization using Jacobian matrices *e*_*p*,*k−h*_, *e*_*l*,*k−g*_, and *e*_*π*,*k−j*_, as shown in Eq (5).


$$F\left(R_{w}^{c_{k}},p_{w}^{c_{k}}\right)={\sum_{h=0}^{H_{2}}\left\|e_{p,k-h}\right\|}_{\Sigma_{p,k-h}}^{2}+{\sum_{g=0}^{G_{2}}\left\|e_{l,k-g}\right\|}_{\Sigma_{l,k-g}}^{2}+{\sum_{j=0}^{J_{2}}\left\|e_{\pi ,k-j}\right\|}_{\Sigma_{\pi ,k-j}}^{2}$$ (5)

In Eq (5), *H*_1_, *G*_1_, and *J*_1_ represent the total number of corresponding points, lines, and surfaces in the matching set. Finally, by minimizing $F(R_{w}^{c_{k}},p_{w}^{c_{k}})$ using the g2o library and LM, a more accurate $\left(R_{c_{k}}^{w},p_{c_{k}}^{w}\right)$ can be obtained. Taking into account the tracking quality of the visual odometer and the situation of backend optimization, a decision is made on whether to insert keyframes. Redundant keyframes can be removed from the map. Then, 3D lines and surfaces in the current frame’s camera coordinate system are extracted to form the matrix $R_{MF}^{c_{k}}$ using three coordinate axis vectors. Additionally, because the direction vectors of lines are not completely perpendicular to the normal vectors of surfaces, singular value decomposition is employed to correct them. Finally, the current frame’s pose is determined using the reference history frames MF, their corresponding $R_{c_{k}}^{w}$, $R_{MF}^{c_{k}}$, and $R_{MF}^{c_{r}}$ [23]. From this, the current frame posture can be obtained, as shown in Eq (6).


$${\tilde{R}}_{c_{k}}^{w}=R_{c_{r}}^{w}•R_{MF}^{c_{r}}•{\left(R_{MF}^{c_{k}}\right)}^{-1}$$ (6)

**Fig 2:**
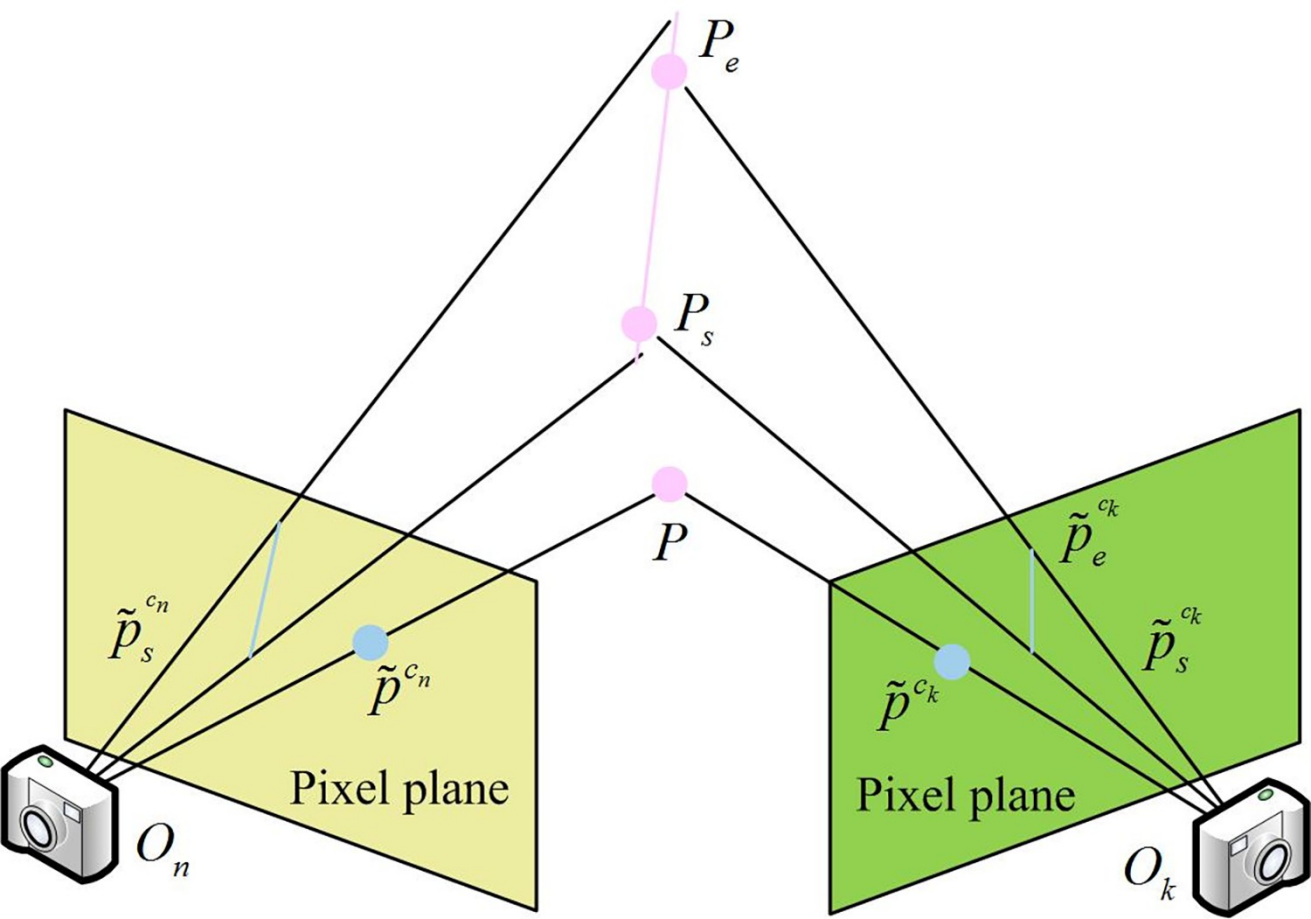
The triangulation process of 2D points and lines.

Updating the local map is required, and the remaining two-dimensional (2D) points and lines in the current frame need to undergo triangulation, as shown in Fig 2.

In Fig 2, point *P* is the intersection of the rays ${\tilde{p}}^{c_{k}}$ and ${\tilde{p}}^{c_{n}}$, both emanating from the optical center *O*_*k*_. The starting point *P*_*s*_ and the endpoint *P*_*e*_ of the map line are the intersections of rays $O_{k}{\tilde{p}}_{s}^{c_{k}}$, $O_{n}{\tilde{p}}_{s}^{c_{n}}$, and the plane *π*’. In the backend optimization, when keyframes arrive, research focuses on introducing measurement error terms corresponding to lines, planes, and MF to construct the objective function, as shown in Eq (7).


$$F(\alpha )=\sum_{k=0}^{K}\left({\sum_{h=0}^{H_{k}}\left\|e_{p,k-h}\right\|}_{\Sigma_{p,k-h}}^{2}+{\sum_{g=0}^{G_{k}}\left\|e_{l,k-g}\right\|}_{\Sigma_{l,k-g}}^{2}+{\sum_{j=0}^{J_{k}}\left\|e_{\pi ,k-j}\right\|}_{\Sigma_{\pi ,k-j}}^{2}+{\left\|e_{MF,k}\right\|}_{\Sigma_{MF,k}}^{2}\right)$$ (7)

In Eq (7), *H*_*k*_, *G*_*k*_, and *J*_*k*_ are the total numbers corresponding to the matching points, lines, and planes in the set of the *k*th keyframe. *e*_*MF*,*k*_ and Σ_*MF*,*k*_ are the measurement error terms and the measurement noise covariance matrix of MF, respectively. Finally, the Jacobian matrix of the measurement error terms corresponding to points, lines, and MF is constructed, and the solution is obtained through the g2o library and LM.

### 3.2. Design of IMS-VSLAM algorithm

**Fig 3:**
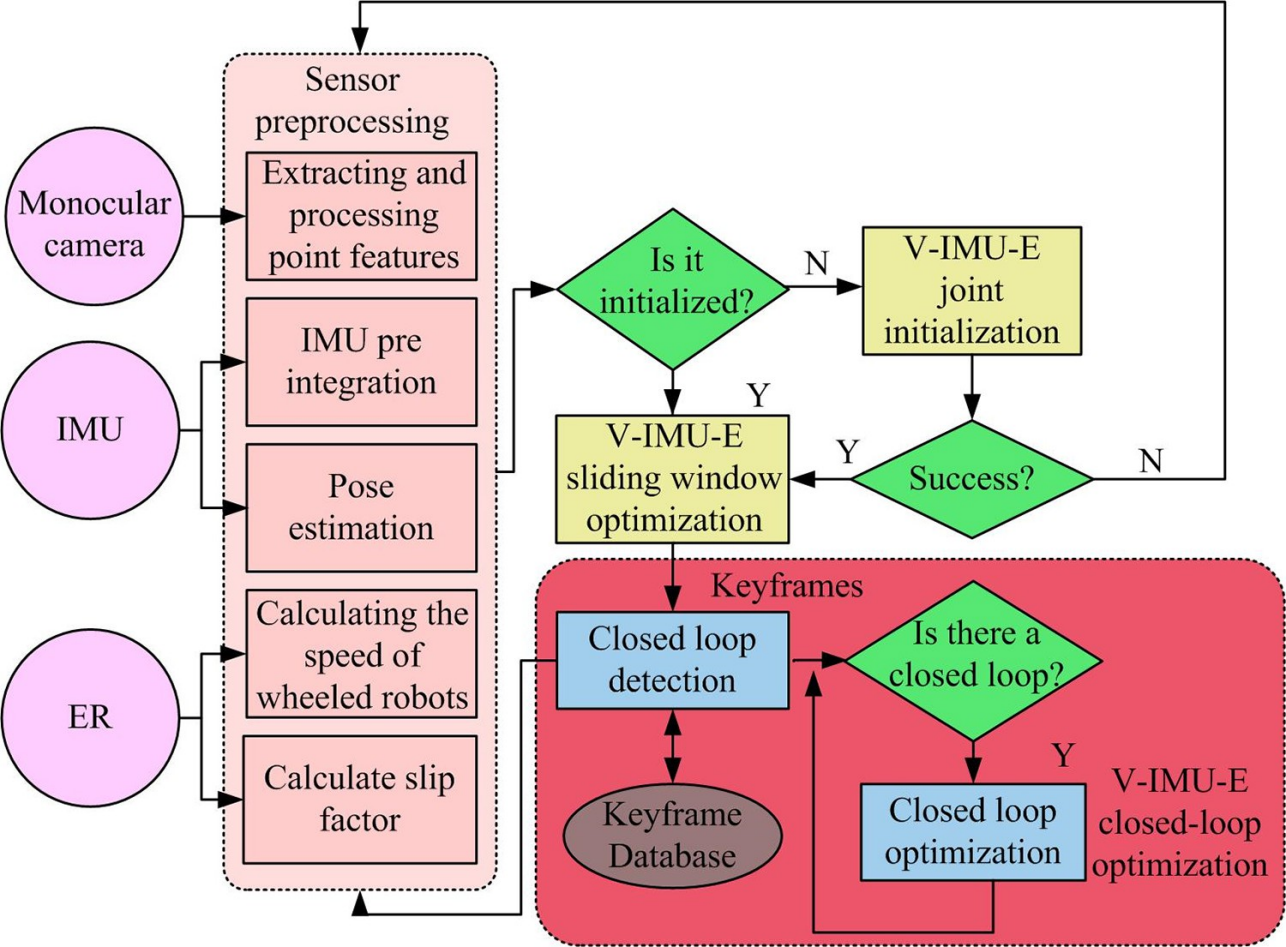
Process diagram of IMS-VSLAM algorithm.

In the face of more complex scenes, the OMFF-SLAM algorithm mentioned above may encounter tracking loss issues. Therefore, this study combines multiple sensors, IMU, and the VIFPO algorithm to obtain the IMS-VSLAM algorithm, with the corresponding flow diagram shown in Fig 3.

Fig 3 illustrates the process diagram of the IMS-VSLAM algorithm, which includes multi-sensor preprocessing, joint initialization processing of vision-IMU-encoder (V-IMU-E), V-IMU-E sliding window optimization, and loop closure optimization. In the visual part of multi-sensor preprocessing, the Kanade-Lucas-Tomasi optical flow method (KLTOF) is used to track Harris point features in adjacent frame images and construct matching relationships to obtain the coordinates $\tilde{p}={\left[\tilde{u},\tilde{v}\right]}^{T}$ of point features in the image [24,25]. The KLTOF method assumes that the grayscale values of objects in the image remain unchanged in two frames, and adjacent pixels have the same motion. Then, based on the principle of optical flow, point features in adjacent images with similar distances and small time intervals are tracked. This method can effectively save computation time and make better use of scenes with sparse features. IMU preprocessing requires first constructing the measurement model of a wheeled robot in discrete time, as shown in Eq (8).


$$\left\{ \begin{array}{l} {\tilde{a}}_{k+q/n}=a_{k+q/n}+R_{w}^{s_{k+q/n}}\cdot b^{w}+s_{a_{k}}+n_{a}\\ {\tilde{\omega }}_{k+q/n}=\omega_{k+q/n}+s_{\omega_{k}}+n_{\omega } \end{array}\right.$$ (8)

In Eq (8), *k*+*q*/*n* is the time corresponding to the *q*th frame IMU between the *k*th and *k*+1 frames, *a*_*k*+*q*/*n*_ A and *ω*_*k*+*q*/*n*_ are the true values of the wheeled robot’s acceleration and angular velocity, $R_{w}^{s_{k+q/n}}$ represents the rotation matrix from the world coordinate system to the time-IMU coordinate system of the *q*th frame, *b*^*w*^ is the gravity vector in the world coordinate system, $s_{a_{k}}$ and $s_{\omega_{k}}$ are the biases corresponding to *a* and *ω* between the *k*th and *k*+1 frames. *n*_*a*_ and *n*_*ω*_ are the Gaussian measurement noises corresponding to *a* and *ω*. Finally, integrating the IMU measurement yields the IMU observation values. Subsequently, the IMU acceleration measurement values are transformed, gravity vector is removed after transforming to the world coordinate system, and the pose of the wheeled robot can be calculated. In the encoder preprocessing, the velocity and slip factor *φ*_*k*_ of the wheeled robot are included, as expressed in Eq (9).


$$\left\{ \begin{array}{l} {\tilde{v}}_{s_{k}}^{s_{k}}=R_{e}^{s}\left[{\tilde{v}}_{e_{k}}^{e_{k}}+\omega_{e_{k}}{\left\{{\left(p_{e}^{s}\right)}_{y}-{\left(p_{e}^{s}\right)}_{x}0\right\}}^{T}\right]\\ \varphi_{k}=\left\{ \begin{array}{l} 0,|\omega_{e_{k}}-\omega_{s_{k}}|>\beta \\ \exp\left[-\frac{{\left(\omega_{e_{k}}-\omega_{s_{k}}\right)}^{2}}{\chi }\right],|\omega_{e_{k}}-\omega_{s_{k}}|\leq \beta \end{array}\right. \end{array}\right.$$ (9)

In Eq (9), $R_{e}^{s}$ E represents the rotation of encoder relative to IMU, and ${\tilde{v}}_{e_{k}}^{e_{k}}$ and ${\tilde{v}}_{s_{k}}^{s_{k}}$ are the IMU velocity observations and encoder velocity, respectively. $\omega_{e_{k}}$ is the angular velocity of the wheeled robot in the encoder coordinate system for the *k*th frame, and ${\left(p_{e}^{s}\right)}_{x}$ and ${\left(p_{e}^{s}\right)}_{y}$ are the *x* and *y* components of the displacement of encoder relative to IMU position $p_{e}^{s}$. $\omega_{e_{k}}$ and $\omega_{s_{k}}$ are the angular velocities of encoder and the instantaneous angular velocity of the wheeled robot computed by IMU, respectively. *χ* is the rate of change of the adjustment variable *φ*_*k*_. *β* represents the threshold of encoder data used to determine its availability. In the V-IMU-E initialization section, initialization is performed for the pose and velocity of the wheeled robot, IMU angular velocity bias, and map point positions. encoder error terms are introduced in the construction of the objective function to improve the accuracy of the estimates. Additionally, to avoid the impact of wheel slippage on the initialization results, *φ*_*k*_ is evaluated, and if its value is too small, initialization is exited. Otherwise, initialization is performed after receiving preprocessed images. In the V-IMU-E sliding window optimization section, the IMS-VSLAM algorithm introduces visual, encoder, and IMU data to construct the objective function, as shown in Eq (10).


$$F'(\alpha )=\sum_{k=0}^{K}\sum_{h=0}^{H_{k}}\rho ({\left\|e_{CA,k-h}\right\|}_{\Sigma_{CA,k-h}}^{2})+\sum_{k=0}^{K-1}{\left\|e_{IMU,k-k+1}\right\|}_{\Sigma_{IMU,k-k+1}}^{2}+\sum_{k=0}^{K}{\left\|e_{ER,k}\right\|}_{\Sigma_{ER,k}/\varphi_{k}}^{2}+{\left\|r_{prior}-H_{prior}•\gamma \right\|}^{2}$$ (10)

In Eq (10), there are the Mahalanobis distance measurement error terms for visual, IMU, and encoder, respectively, representing the marginalization term. H is the total number of maps for the Kth frame, P is the Huber robust kernel function, and E, EI, and EG are the noise covariance matrices for visual, IMU, and encoder, respectively, representing the state variables. Finally, the Jacobian matrices of the measurement error terms corresponding to visual, IMU, and encoder are constructed, obtained through the Ceres library and LM. The pose of the wheeled robot is the localization result in an open-loop environment, and the current frame is judged for insertion into subsequent loop closure optimization based on the average parallax of the tracked feature points. In the V-IMU-E loop closure optimization section, to eliminate the accumulated error in wheeled robot pose estimation during the V-IMU-E window optimization process, the algorithm uses loop closure detection and optimization with VIFPO [26,27]. The specific process is as follows: first, loop closure detection is performed on the ORB features of keyframes transmitted from the V-IMU-E window optimization section. If a loop is detected, optimization steps can be taken. In the loop closure optimization, the gravity vector obtained from IMU is aligned with the Earth to build the world coordinate system. The optimization focuses on the position and heading angle of the wheeled robot for keyframes, expressing the state variables as shown in Eq (11).


$$\gamma_{lp}=[p_{s_{0}}^{w},p_{s_{1}}^{w},\cdots ,p_{s_{K'}}^{w},\varepsilon_{s_{0}}^{w},\varepsilon_{s_{1}}^{w},\cdots ,\varepsilon_{s_{L}}^{w}]$$ (11)

In Eq (11), *k*’ represents the total number of keyframes in the loop closure, and $p_{s_{k}}^{w}$ and $\varepsilon_{s_{k}}^{w}$ are the position and heading angle of the wheeled robot in the world coordinate system for the *k*th frame. The calculation of robot position observations for the *i*th and *j*th frames is given by Eq (12).


$${\tilde{p}}_{s_{j}}^{s_{i}}=\frac{p_{s_{j}}^{w}-p_{s_{i}}^{w}}{R_{s_{i}}^{w}}$$ (12)

The calculation of the heading angle for the two frames is given by Eq (13).


$${\tilde{\varepsilon }}_{s_{j}}^{s_{i}}=\varepsilon_{s_{j}}^{w}-\varepsilon_{s_{i}}^{w}$$ (13)

By establishing error terms based on the observations, the formulation is given in Eq (14).


$$e_{i,j}=[\begin{array}{l} {\tilde{p}}_{s_{j}}^{s_{i}}-(p_{s_{j}}^{w}-p_{s_{i}}^{w})•{\left(R_{s_{i}}^{w}\right)}^{-1}\\ {\tilde{\varepsilon }}_{s_{j}}^{s_{i}}-\varepsilon_{s_{j}}^{w}+\varepsilon_{s_{i}}^{w} \end{array}]$$ (14)

Finally, the objective function is constructed as shown in Eq (15).


$$F\left(\alpha_{lopp}\right)={\sum_{i,j\in S}\left\|e_{i,j}\right\|}^{2}+\sum_{i,j\in L}\rho \|e_{i,j}^{2}\|$$ (15)

In Eq (15), *S* and *L* are the sets of fixed interval two frames and their corresponding sets in the loop closure. The result of wheeled robot localization corresponding to loop closure detection is obtained by solving *F*(*α*_*lopp*_) using LM.

### 3.3. Construction of wheeled robot platform

**Fig 4:**
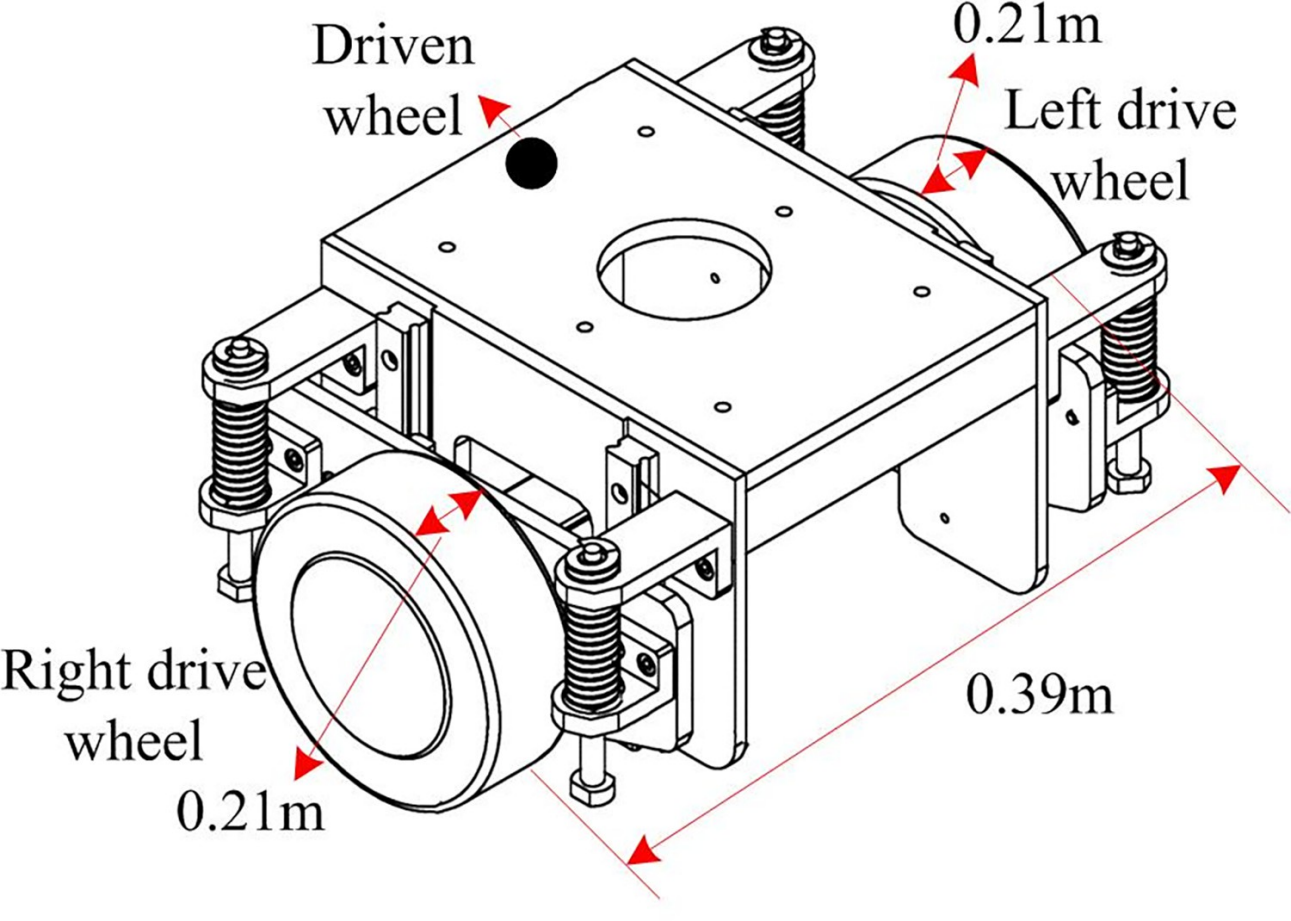
Structural model of TWR robot.

Wheeled robots, capable of intelligently performing various tasks in industrial and other fields, significantly enhance work efficiency and substantially reduce labor costs. They can work continuously for long periods without the need for rest, making them widely applicable in various domains and holding significant importance for modern production and life [28]. The study involves constructing a two-wheeled robot and platform to assess the algorithm’s performance and application effects proposed in the research. The TWR structural model developed in the study is depicted in Fig 4.

**Fig 5:**
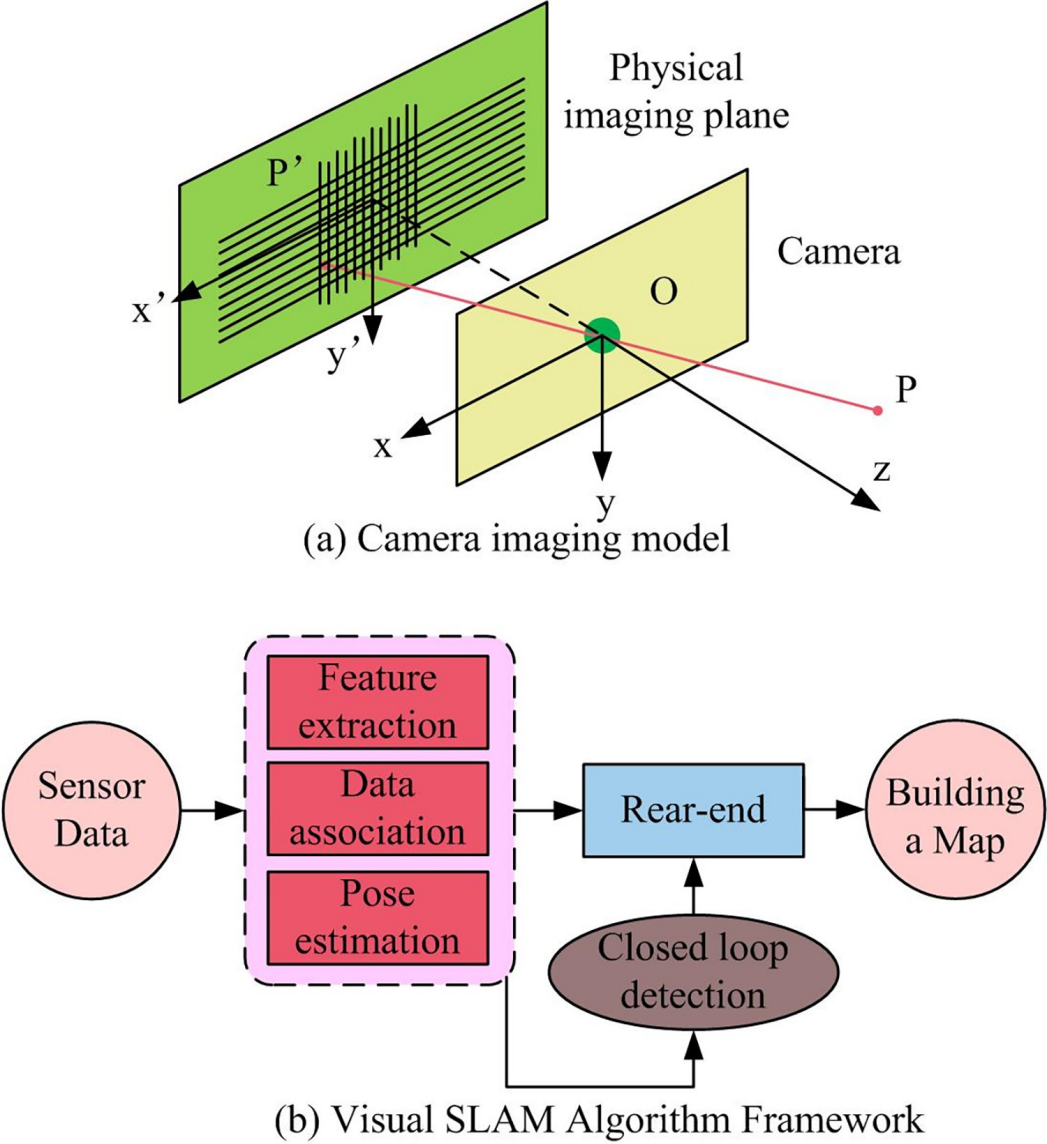
The framework of camera imaging model and visual SLAM algorithm.

In Fig 4, the front wheels consist of two independently driven wheels used to change the speed, direction, and size, enabling the TWR robot to move along the planned path. The rear wheels serve as passive wheels to stabilize the TWR robot. The TWE robot’s dimensions and mass are set at 0.46m, 0.48m, 0.61m, and 25kg, respectively. Laser radar and a computer support axle are installed at the front and rear, respectively, to adjust the RealSense D435i camera’s field of view. The camera imaging model and the framework of the visual SLAM algorithm are illustrated in Fig 5.

**Fig 6:**
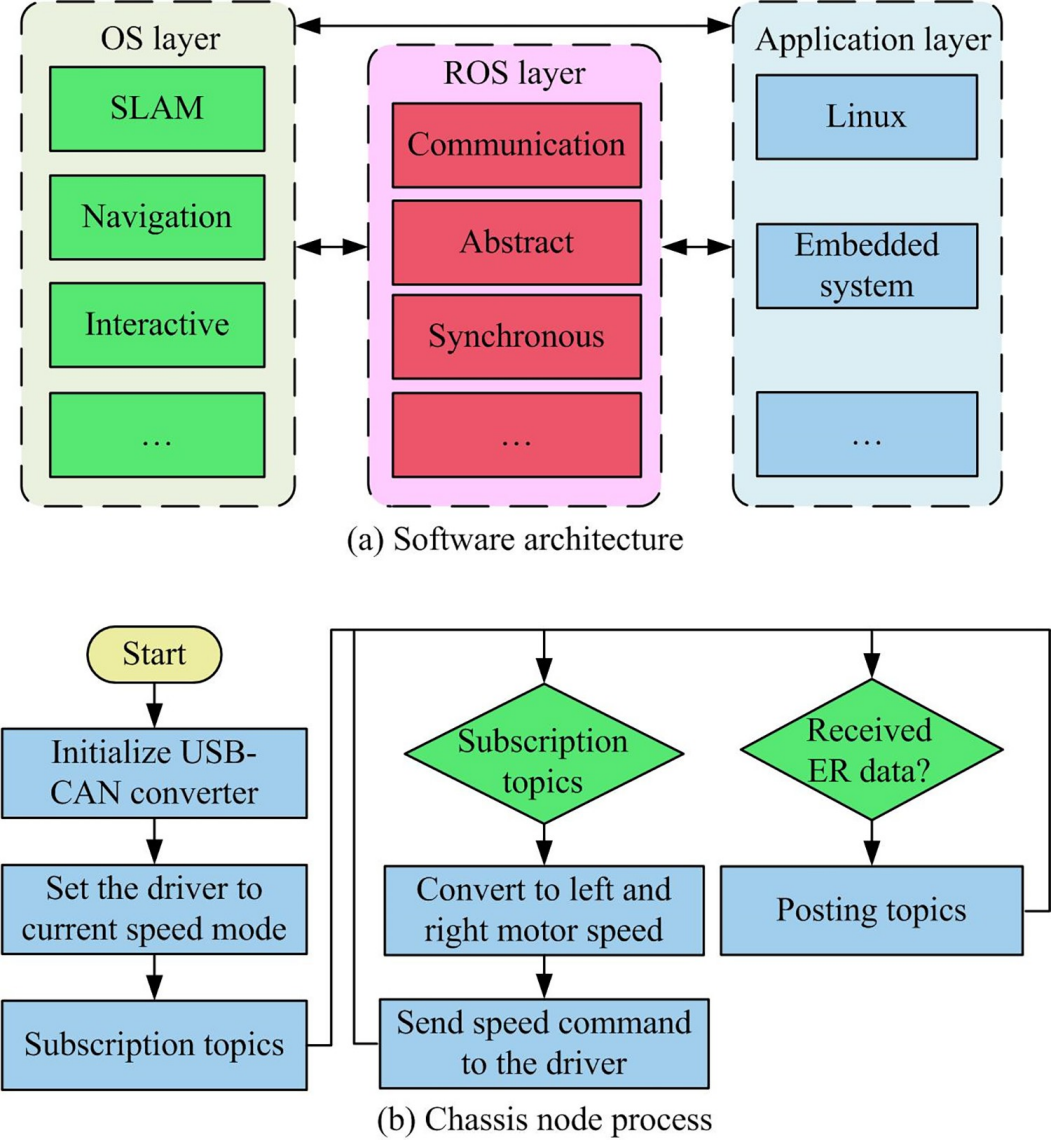
Schematic diagram of software architecture and chassis node program flow for wheeled robot platform.

In Fig 5, the camera’s imaging model represents the correspondence between real-world 3D points and 2D image pixels. Under the assumption of no distortion, the ideal model for monocular camera imaging is the pinhole model. The optical axis projects through the optical center and is perpendicular to the physical imaging plane, with each 3D point in space corresponding to a pixel on the physical imaging plane. Classical visual SLAM algorithms consist of five parts: sensor data acquisition, visual odometry, backend, loop closure detection, and map construction. The LiDAR is used to collect point cloud data. In the subsequent performance testing using data from the TUM dataset, it is necessary to run the LiDAR point cloud data with the camera’s IMU data to obtain the true trajectory and speed. Finally, the software for the TWR robot platform is developed, as detailed in Fig 6.

In Fig 6, the software includes the Operating System layer (OS), the Robot Operating System layer (ROS), and the application layer. The OS layer comprises the installed Linux system and the embedded system corresponding to the microcontroller, managing the device’s software and hardware. The ROS layer provides communication, time synchronization, and other functions, while the application layer includes SLAM, interaction, etc. The bottom-level node process involves initializing the USB-CAN converter, sending commands to the driver to set it to current velocity mode, and finally publishing relevant topics through the joystick node.

## 4. Analysis of IMS-VSLAM algorithm for wheeled robots

To evaluate the performance and application effects of the proposed IMS-VSLAM algorithm, experiments were conducted on the constructed TWR robot platform. The study began with an analysis of the performance of the OMFF-SLAM algorithm, followed by an exploration of the IMS-VSLAM algorithm’s performance and application effects in different environments.

### 4.1. Results analysis based on OMFF-SLAM algorithm

To investigate the performance of the OMFF-SLAM algorithm, the study utilized the commonly used TUM dataset in visual SLAM research. This dataset comprised 39 indoor sequences captured under varying conditions of texture, lighting, and structure. Data, acquired through RGB-D sensors, were employed to assess the reconstruction of objects and the performance. Additionally, the dataset captured objects from favorable viewpoints, with each entry containing image sequences, corresponding contours, and complete calibration parameters. For a more scientifically rigorous assessment of the proposed algorithm’s performance, the study conducted comparative experiments with mainstream SLAM algorithms, including Improved SLAM Based on LiDAR (LiDARO-SLAM) and SLAM Based on the Fusion of Binocular Wire Features and Inertial Navigation (BWFIN-SLAM) [29,30].

**Fig 7:**
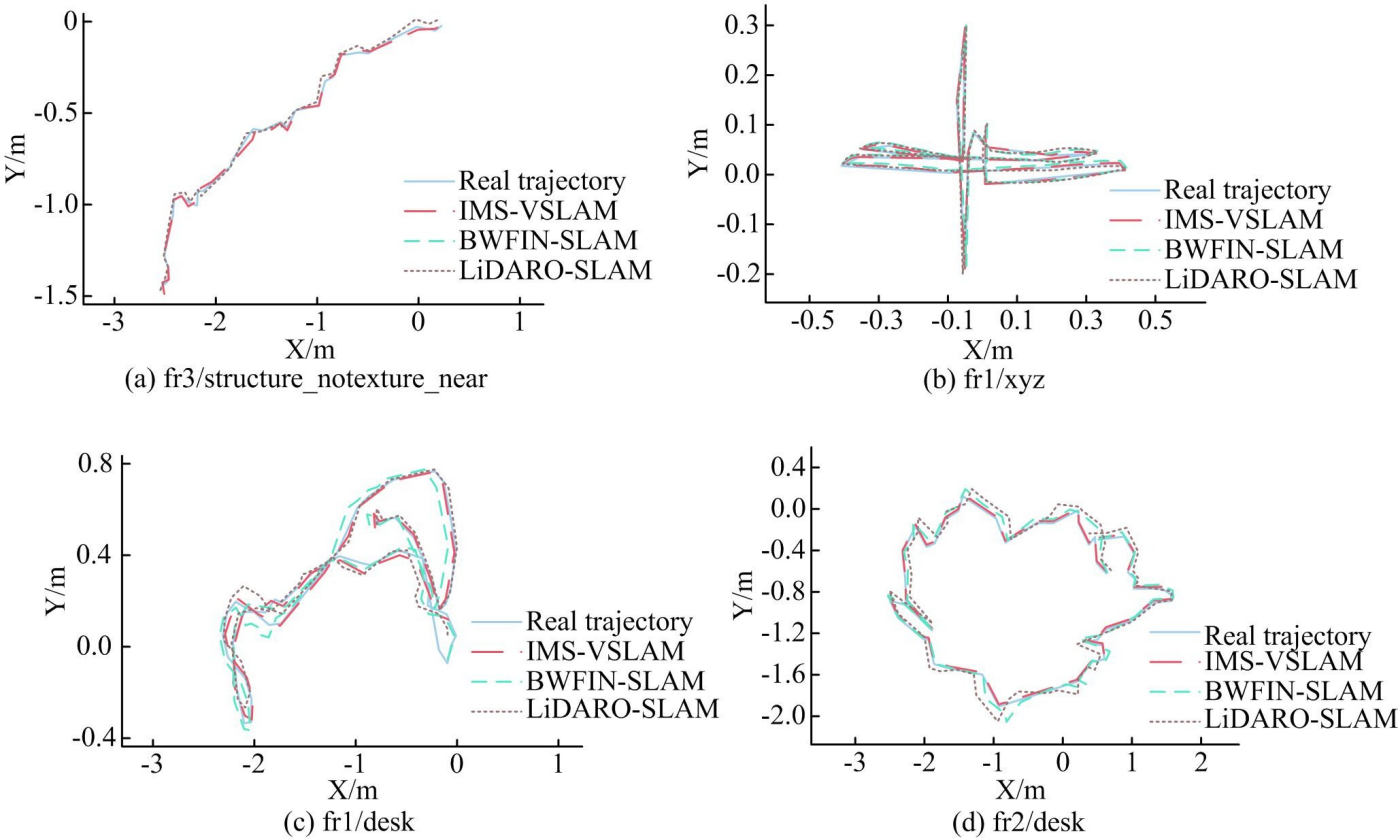
Comparison of positioning accuracy results of different algorithms based on the TUM dataset.

Fig 7(A) to 7(D) depicted the localization accuracy results of different algorithms for low-texture data packages fr3/structure_notexture_near, fr1/xyz, fr1/desk, and fr2/desk. From Fig 7, it could be observed that the BWFIN-SLAM algorithm, due to the insufficient extraction of point features, experienced tracking losses leading to tracking failures. On the other hand, both LiDARO-SLAM and OMFF-SLAM algorithms, incorporating line and plane features, contributed to an improvement in the trajectory estimation success rate for the TWR robot. To further quantify the algorithmic errors, the study employed the Root Mean Square Error (RMSE) for evaluation. Additionally, the real-time performance of the algorithms was assessed through the Average Time (AT) taken to process a single frame image. The results were summarized in Table 2.

**Table 2:** Performance comparison of different algorithms based on TUM dataset.

Data packet	Duration/s	OMFF-SLAM algorithm			BWFIN-SLAM algorithm		LiDARO-SLAM algorithm		
		RMSE/m	MF quantity	AT/ms	RMSE/m	AT/ms	RMSE/m	MF quantity	AT/ms
fr1/desk	23.40	0.032	0	61.9	0.025	41.2	0.030	1	76.2
fr1/xyz	30.09	0.009	4		0.008		0.007	3	
fr2/xyz	122.74	0.003	116		0.008		0.012	0	
fr2/desk	99.36	0.022	46		0.024		0.023	24	
fr3/nostructure_texture_far	15.53	0.010	636		0.010		0.020	576	
fr3/structure_notexture_far	27.28	0.018	645		Fail		0.046	678	
fr3/structure_texture_near	36.91	0.008	574		0.010		0.015	568	
fr3/structure_notexture_near	36.44	0.023	687		Fail		0.024	797	
fr3/large_cabinet	33.98	0.048	469		Fail		0.076	195	

From Table 2, the RMSE values of the OMFF-SLAM algorithm and the BWFIN-SLAM algorithm were very close, with the former exhibiting an average accuracy improvement of 2.12%. In comparison to the LiDARO-SLAM algorithm, the OMFF-SLAM algorithm demonstrated a significant average accuracy improvement of 27.05%. Across the data packages fr3/nostructure_texture_far, fr3/structure_notexture_far, fr3/structure_texture_near, and fr3/structure_notexture_near, the OMFF-SLAM algorithm and the LiDARO-SLAM algorithm showed minimal differences in the number of Map Features (MFs), with slight variations in the remaining data packages. This could be attributed to the OMFF-SLAM algorithm’s requirement for a single surface in its field of view, whereas the LiDARO-SLAM algorithm necessitated the presence of two or more surfaces. Consequently, the proposed algorithm could capture more features through line and surface features. In terms of real-time performance, the OMFF-SLAM algorithm, BWFIN-SLAM algorithm, and LiDARO-SLAM algorithm exhibited AT values of 61.9ms, 41.1ms, and 75.3ms, respectively. This might be attributed to the additional time required by the OMFF-SLAM and LiDARO-SLAM algorithms to process line and surface features. However, the algorithm proposed in the study, utilizing FLD keypoints, demonstrated superior real-time performance. To investigate the effectiveness of the OMFF-SLAM algorithm applied to wheeled robots, the study employed a TWR robot to collect data in indoor environments with varying feature densities: Regular Indoor (RI) and Sparse Indoor Features (SIFs). The camera’s optical axis relative to the ground was set at angles of 33° and 42°, corresponding to the two datasets. The collected data included three trajectories each for RI and SIF datasets. They were in_ ge_ quad, in_ ge_ splay, and in_ ge_ twist; in_ sp_ quad, in_ sp_ splay, and in_ sp_ twist. The results, as shown in Table 3, compared the performance of different algorithms in various indoor datasets.

**Table 3:** Comparison of performance results of different algorithms on different indoor datasets.

Algorithm	Evaluating indicator	Data packet					
		in_ge_quad	in_ge_splay	in_ge_twist	in_sp_quad	in_sp_splay	in_sp_twist
/	Duration/s	117.42	68.75	140.04	143.56	125.07	106.98
OMFF-SLAM	RMSE/m	0.139	0.160	0.164	0.172	0.176	0.181
	MFs quantity	73	214	206	136	257	243
	AT/ms	73.6					
BWFIN-SLAM	RMSE/m	0.159	0.176	0.187	0.192	0.205	0.223
	AT/ms	48.7					
LiDARO-SLAM	RMSE/m	0.178	0.187	0.193	0.208	0.224	0.237
	MFs quantity	0	3	7	3	24	97
	AT/ms	86.2					

From Table 3, it was evident that in the RI dataset, the OMFF-SLAM algorithm achieved the highest average accuracy, surpassing the BWFIN-SLAM and LiDARO-SLAM algorithms by 9.83% and 16.34%, respectively. Additionally, the OMFF-SLAM algorithm had 483 more MFs compared to the LiDARO-SLAM algorithm. In the SIF dataset, the OMFF-SLAM algorithm maintained the highest average accuracy, outperforming the BWFIN-SLAM and LiDARO-SLAM algorithms by 3.04% and 4.67%, respectively. Regarding real-time performance, the AT values for OMFF-SLAM, BWFIN-SLAM, and LiDARO-SLAM were 73.6ms, 48.7ms, and 86.2ms, respectively. Compared to LiDARO-SLAM, OMFF-SLAM demonstrated an improvement of 12.6ms. These results indicated that the application of the three algorithms in the SIF dataset was less effective, leading to instances of tracking loss. In summary, the OMFF-SLAM algorithm exhibited excellent performance, laying a solid foundation for the IMS-VSLAM algorithm.

### 4.2. Analysis of IMS-VSLAM algorithm results for wheeled robots

To assess the performance and application effectiveness of the IMS-VSLAM algorithm, the study initially conducted a slip verification experiment to analyze the algorithm’s slip detection capabilities. During this experiment, the field of view angle of the TWR robot was adjusted, setting the camera’s optical axis inclination relative to the ground at 42°. Subsequently, six instances of artificially generated slip trajectories were collected, and the data package "in_sp_slip" was uploaded.

**Fig 8:**
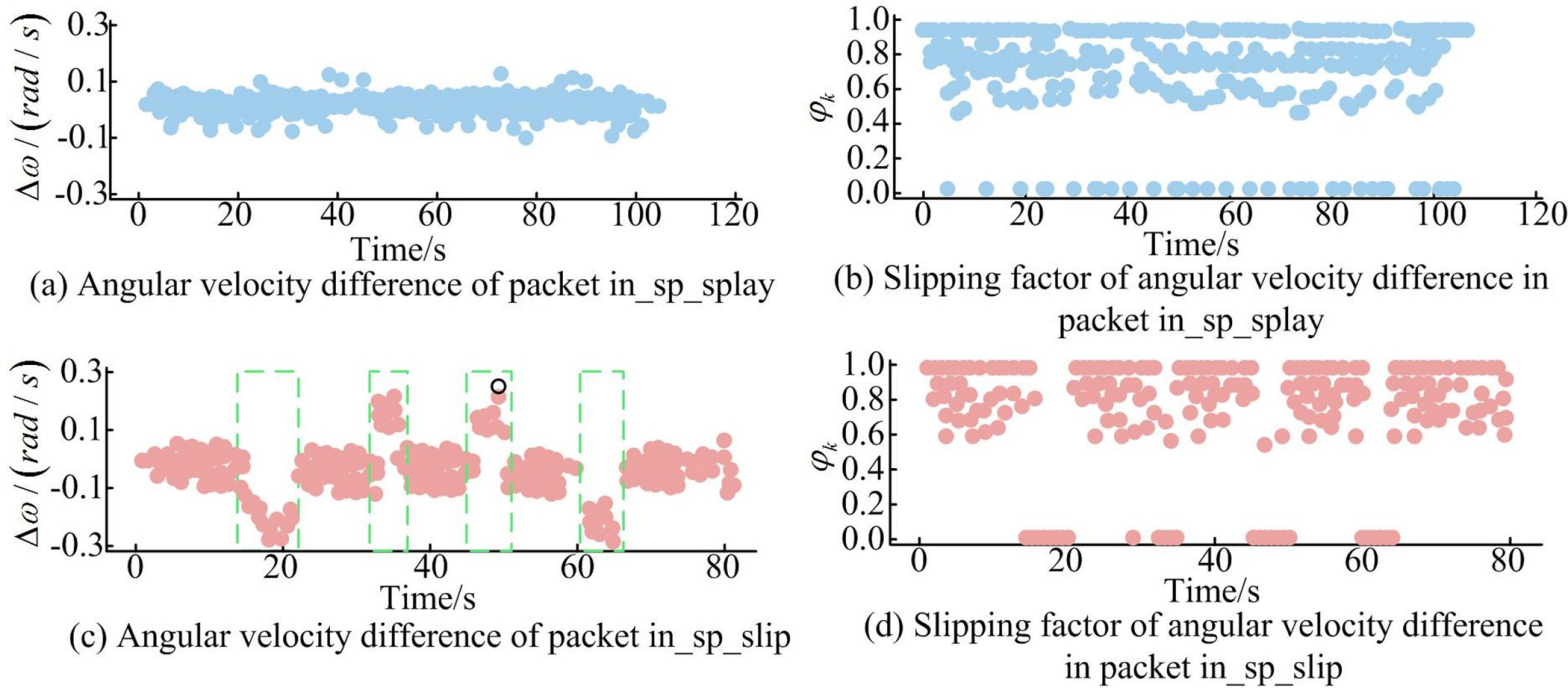
Experimental results of slip based on IMS-VSLAM algorithm under different data packets.

Fig 8(A)–8(D) depicted the results of the IMS-VSLAM algorithm in terms of angular velocity differences and *φ*_*k*_ for the data package "in_sp_splay" and angular velocity differences and *φ*_*k*_ for the data package "in_sp_slip." From Fig 8, it could be observed that in the results for the "in_sp_splay" data package, angular velocity differences fluctuated around 0.1 rad/s, and *φ*_*k*_ varied in the range of 0.5 to 1.0. In the TWR robot slip data package "in_sp_slip," only the four marked green boxes contained a significant number of points where angular velocity differences exceeded 0.1 rad/s, with the *φ*_*k*_ value corresponding to the red box being 0, and the angular velocity difference exceeding *β*. These results indicated that the proposed algorithm could accurately detect slipping in slip experiments. To explore the application effectiveness of the IMS-VSLAM algorithm in indoor scenarios, the study conducted comparative experiments using the RI dataset and SIF dataset. Due to the slow initialization of data packets in the RI dataset or instances of tracking failure in the VIFPO algorithm, the study focused on comparative experiments involving the OMFF-SLAM algorithm, IMS-VSLAM algorithm with closed-loop detection (WCLD-IMS-VSLAM), and IMS-VSLAM algorithm without closed-loop detection (WOCLD-IMS-VSLAM). The performance results of different algorithms based on the RI dataset were presented in Table 4.

**Table 4:** Performance results of different algorithms based on the RI dataset.

Algorithm	Evaluating indicator	Data packet		
		in_sp_quad	in_sp_splay	in_sp_twist
/	Duration/s	117.42	68.75	140.04
WCLD-IMS-VSLAM	RMSE/m	0.132	0.149	0.166
	Running time/ms	96.3	87.8	97.9
	AT/ms	94.0		
WOCLD-IMS-VSLAM	RMSE/m	0.143	0.161	0.167
	Running time/ms	71.9	70.2	75.8
	AT/ms	72.63		
OMFF-SLAM	RMSE/m	0.165	0.176	0.182
	Running time/ms	61.3	61.7	68.2
	AT/ms	63.73		

According to Table 4, WCLD-IMS-VSLAM achieved the highest average accuracy, surpassing the WOCLD-IMS-VSLAM algorithm and OMFF-SLAM by 0.8% and 2.5%, respectively. In terms of real-time performance, the OMFF-SLAM algorithm demonstrated better real-time capabilities, being 30.27ms and 8.90ms faster than the WCLD-IMS-VSLAM and WOCLD-IMS-VSLAM algorithms, respectively. However, the WCLD-IMS-VSLAM algorithm, due to its closed-loop optimization process, required more computational resources, with only a 21.37ms increase in runtime compared to the WOCLD-IMS-VSLAM algorithm. These results suggested that the proposed IMS-VSLAM algorithm with closed-loop detection exhibited more efficient and accurate performance. In the SIF dataset, the OMFF-SLAM algorithm experienced tracking failures for data packets, leading the study to focus comparison experiments solely on the WCLD-IMS-VSLAM algorithm, WOCLD-IMS-VSLAM algorithm, and VIFPO algorithm.

**Fig 9:**
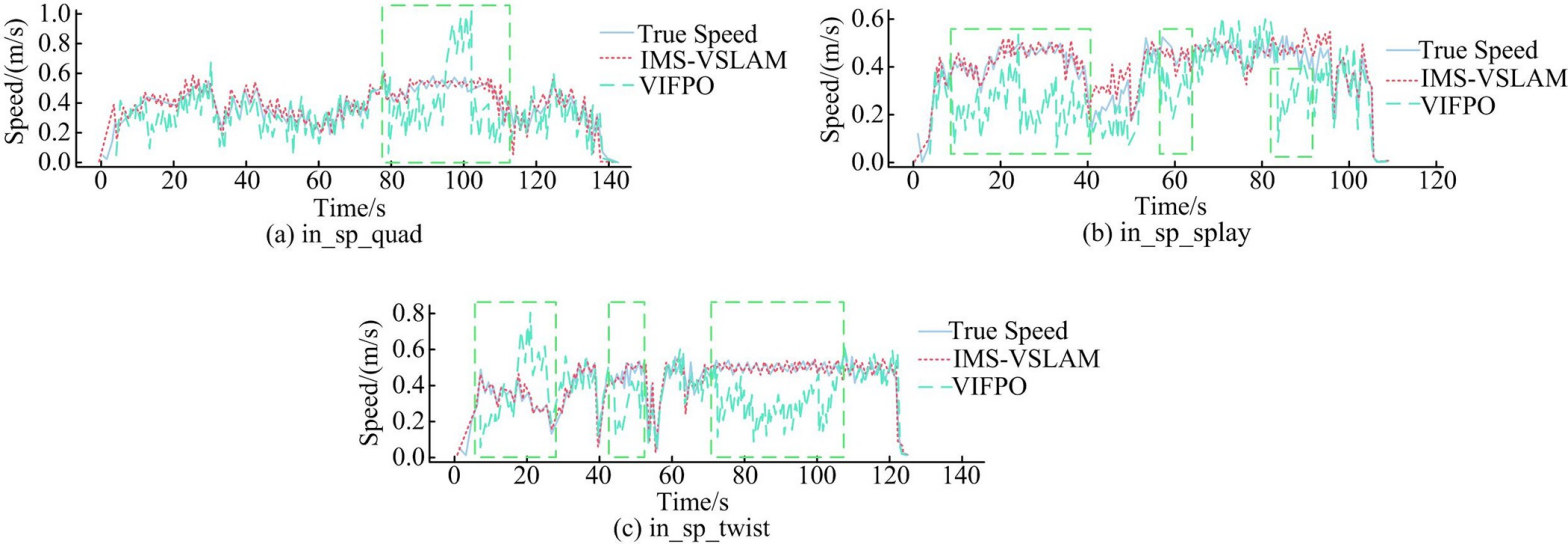
Speed variation curves of different algorithms based on the SIF dataset.

Fig 9(A)–9(C) depicted velocity variation curves for different algorithms under data packets in_sp_quad, in_sp_splay, and in_sp_twist in the SIF dataset. Fig 9 illustrated that, overall, the estimated velocity of the IMS-VSLAM algorithm closely matched the true velocity, while the VIFPO algorithm showed significant discrepancies between estimated and true velocities during periods of constant motion. These results indicated that, under low acceleration conditions, the VIFPO algorithm might have produced significant errors in estimated velocity due to the low signal-to-noise ratio of the IMU accelerometer. To further quantify errors, the study conducted experiments on the performance of different algorithms based on the SIF dataset. To mitigate the impact of V-IMU-E window optimization and closed-loop optimization on runtime, real-time performance metrics excluded these processes, as shown in Table 5.

**Table 5:** Performance results of different algorithms based on SIF dataset.

Algorithm	Evaluating indicator	Data packet		
		in_sp_quad	in_sp_splay	in_sp_twist
/	Duration/s	143.56	125.07	106.98
WCLD-IMS-VSLAM	RMSE/m	0.148	0.139	0.193
	Running time/ms	35.3	37.2	32.0
	AT/ms	34.83		
WOCLD-IMS-VSLAM	RMSE/m	0.179	0.167	0.235
VIFPO	RMSE/m	2.442	4.192	3.103
	Running time/ms	33.3	35.2	33.6
	AT/ms	34.03		

Table 5 revealed that both WCLD-IMS-VSLAM and WOCLD-IMS-VSLAM algorithms had achieved an order of magnitude improvement in average accuracy compared to the VIFPO algorithm. Moreover, WCLD-IMS-VSLAM exhibited a 14.38% increase in average accuracy compared to WOCLD-IMS-VSLAM. In terms of real-time performance, the IMS-VSLAM algorithm’s additional time (AT) was only 0.8ms more than the VIFPO algorithm, indicating that the proposed IMS-VSLAM algorithm effectively retained the excellent real-time performance of the VIFPO algorithm. To further analyze the application effectiveness of the IMS-VSLAM algorithm in outdoor environments, the study selected the outdoor campus of L University as the data collection site. The TWR robot’s field of view angle had been set at a camera optical axis inclination of 33° relative to the ground. Subsequently, outdoor data sets (OD) were collected, including od_spacious, od_3D, and od_cuboid, corresponding to circumnavigating a teaching building, ascending a 3m staircase from the teaching building entrance, and circumnavigating an experimental building, respectively.

**Fig 10:**
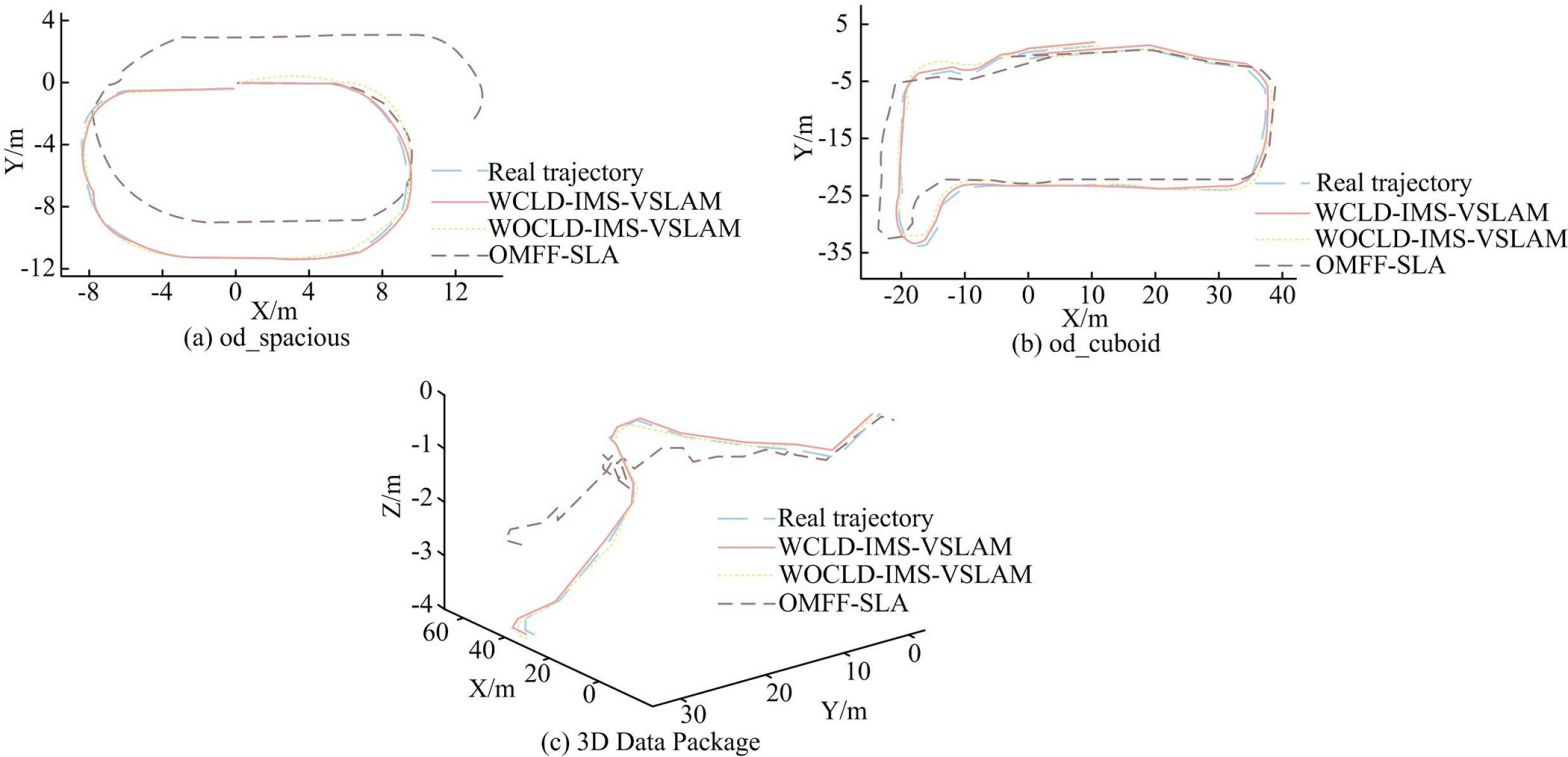
Different algorithm trajectory results based on OD dataset 2D and 3D packets.

Fig 10(A)–10(C) illustrates the trajectory variations of od_spacious, od_cuboid, and different algorithms for 2D and 3D data packets. In Fig 10, it could be observed that in the 2D data packet, due to the spacious outdoor environment where many objects were positioned beyond the camera’s depth measurement threshold, resulting in numerous pixels in the depth map without depth values. This phenomenon led to significant drift in the trajectory of the OMFF-SLAM algorithm. In contrast, the IMS-VSLAM algorithm, which did not rely on depth maps from the camera, exhibited a trajectory estimate that was essentially consistent with the true trajectory. In outdoor scenarios where objects were closer to the camera, the drift in the estimated trajectory of the OMFF-SLAM algorithm decreased noticeably, although it still fell short of the accuracy achieved by the IMS-VSLAM algorithm. In the case of 3D data packets, the IMS-VSLAM algorithm maintained a substantial overlap between the estimated trajectory and the true trajectory. To further quantify the errors and real-time performance of different algorithms in the OD dataset, experiments were conducted, and the results are presented in Table 6.

**Table 6:** Performance results of different algorithms based on OD dataset.

Algorithm	Evaluating indicator	Data packet		
		od_spacious	od_3D	od_cuboid
/	Duration/s	143.92	265.15	399.256
WCLD-IMS-VSLAM	RMSE/m	0.246	0.261	0.257
	Running time/ms	86.8	95.8	92.3
	AT/ms	91.63		
WOCLD-IMS-VSLAM	RMSE/m	0.283	0.315	0.301
	Running time/ms	62.7	73.3	74.3
	AT/ms	70.10		
OMFF-SLAM	RMSE/m	2.55	1.23	1.36
	Running time/ms	60.4	63.2	64.6
	AT/ms	62.73		

From Table 6, it is evident that the IMS-VSLAM algorithm exhibited a significantly higher level of accuracy compared to the OMFF-SLAM algorithm, with the WCLD-IMS-VSLAM algorithm showing a 14.51% improvement in average precision over the WOCLD-IMS-VSLAM algorithm. Regarding real-time performance, the AT values for the WCLD-IMS-VSLAM algorithm, WOCLD-IMS-VSLAM algorithm, and OMFF-SLAM algorithm were 91.63ms, 70.10ms, and 62.73ms, respectively. In summary, the OMFF-SLAM algorithm demonstrated superior accuracy and real-time performance in indoor environments, specifically on the RI dataset. On the other hand, in indoor environments using the SIF dataset and outdoor environments, the IMS-VSLAM algorithm outperformed, displaying better performance and application effectiveness, along with robustness.

### 4.3. Analysis of mobile scenes and snow road effects based on IMS-VSLAM algorithm

To test the effectiveness of the proposed fusion algorithm in scenarios with moving obstacles and complex snowy road scenes, a comparative experiment was conducted using the fusion algorithms from references [8,11]. The study first set up a scene where a person was moving at a constant speed in a straight line (Dynamic Scene 1) for experimentation.

**Fig 11:**
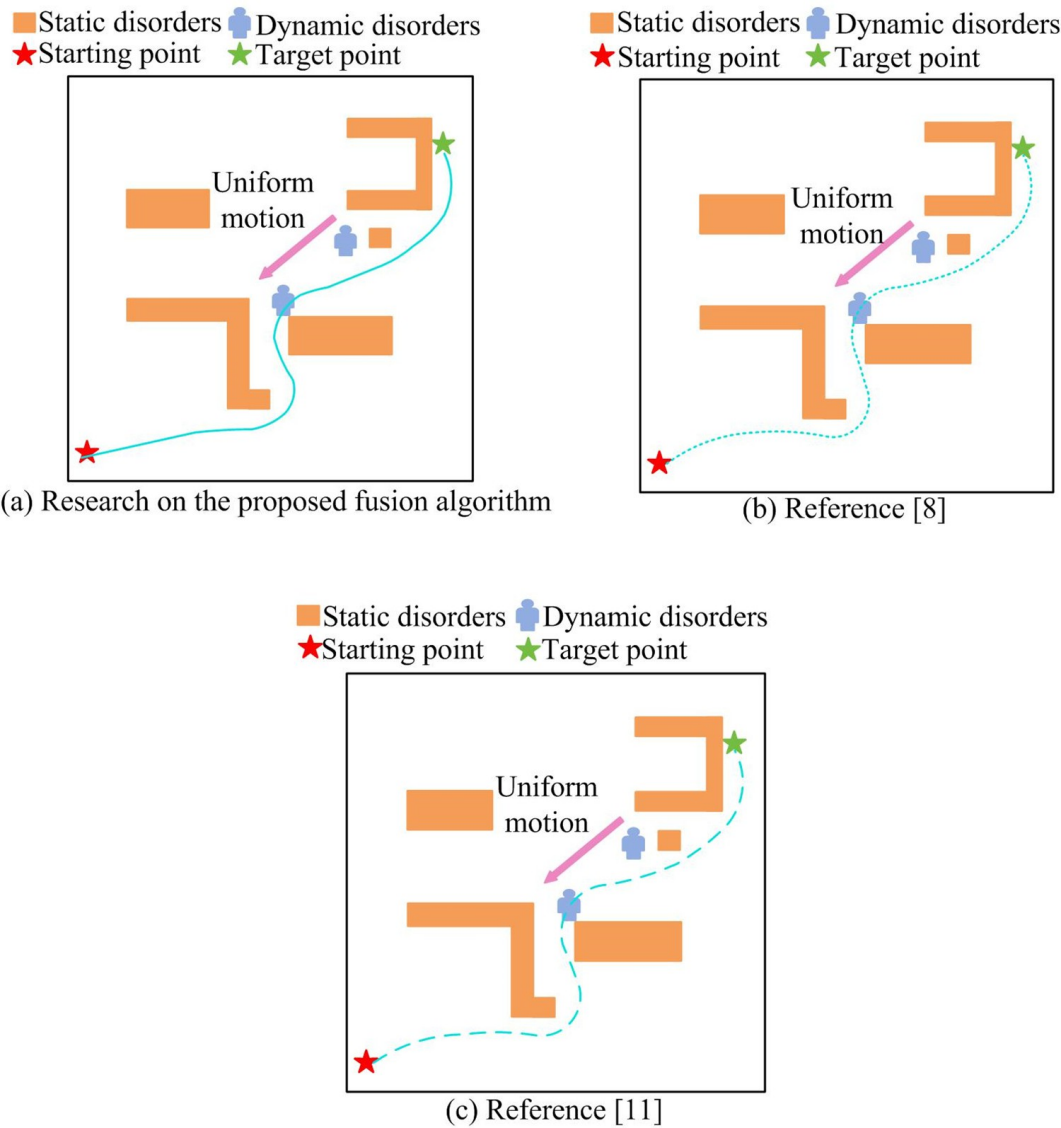
Simulation results of different algorithms in dynamic scenario 1.

Fig 11(A)–11(C) respectively show the simulation results of the proposed fusion algorithm, literature [8], and literature [11] in dynamic scene 1. It could be observed that during the process of human movement, the proposed fusion algorithm quickly adjusted the direction of motion after detecting a person and successfully bypassed the person to reach the endpoint. However, the fusion algorithm used in references [8,11] consistently kept the mobile robot in a small range, making it extremely prone to collisions with humans. In the setting of Dynamic Scene 2, there was a car accelerating.

**Fig 12:**
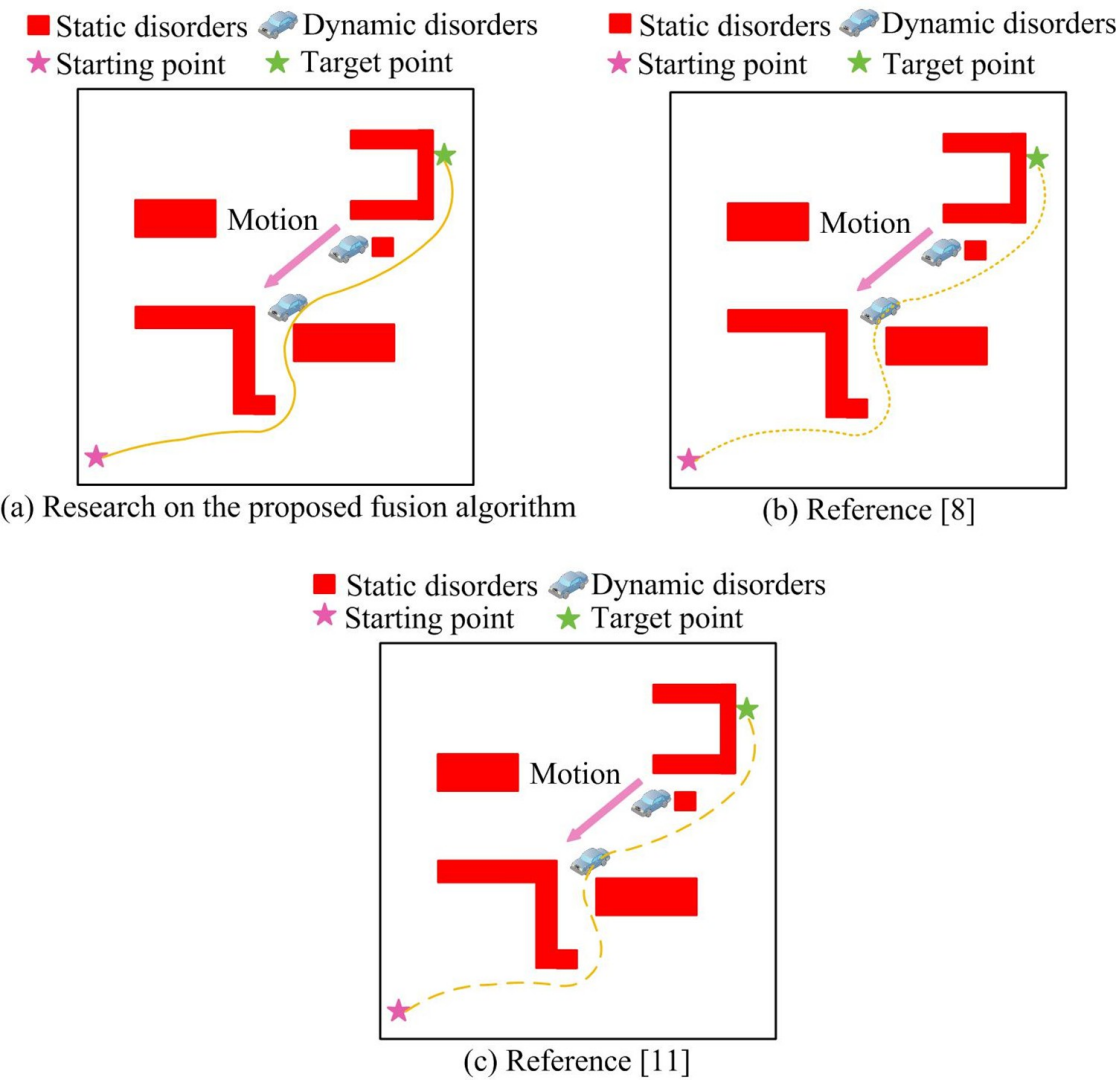
Simulation results of different algorithms in dynamic scenario 2.

Fig 12(A)–12(C) show the simulation results of the proposed fusion algorithm, literature [8], and literature [11] in dynamic scenario 2, respectively. From Fig 12, it could be seen that the fusion algorithm proposed in the study could successfully avoid dynamic car movements and reach the endpoint. Although the other two algorithms had the ability to dynamically avoid obstacles, they did not successfully reach the endpoint. From the overall effect observation, the proposed fusion algorithm had smaller path jitter, smoother curves, and was more conducive to robot control. Finally, the experiment was conducted on a snowy road outside the teaching building, with a static environment and unknown obstacles on the road. A total of 20 experiments were conducted, and the deviation of the driving route of the wheeled robot running through fusion algorithms was analyzed.

**Fig 13:**
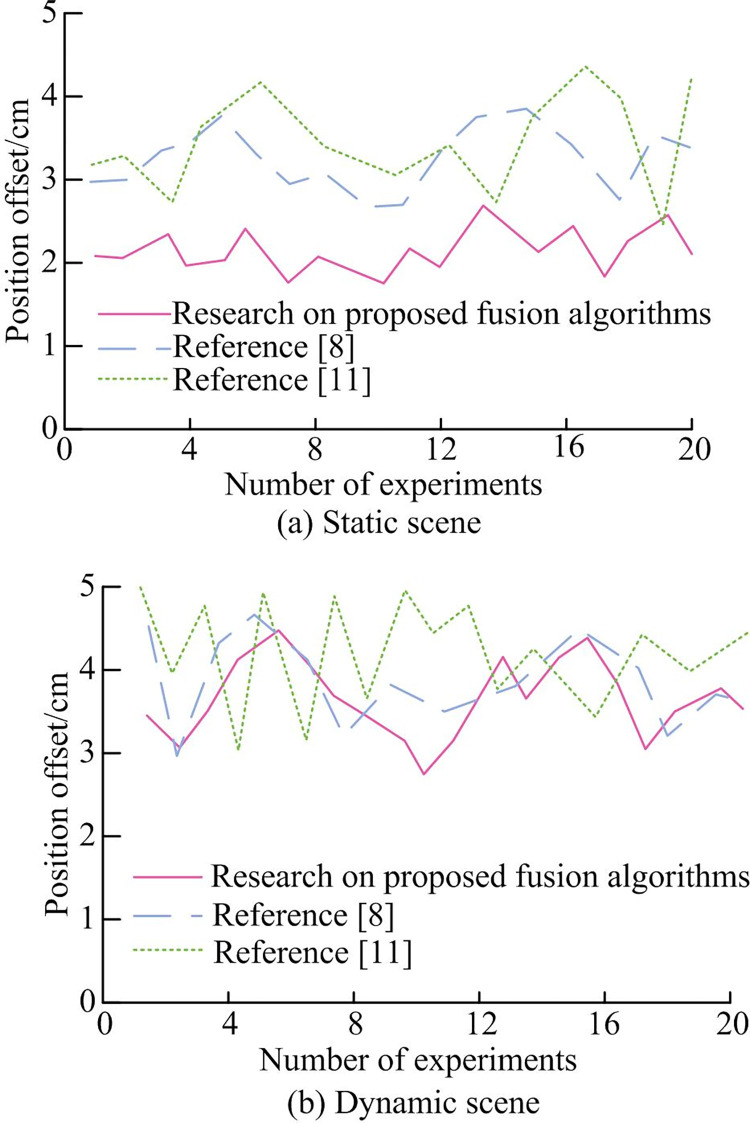
Analysis of the application effects of different fusion algorithms in static scenes and snowy road scenes with unknown dynamic obstacles.

Fig 13(A) and 13(B) show the application performance analysis of different fusion algorithms in static scenes and snowy road scenes with unknown dynamic obstacles, respectively. As shown in Fig 13, the fusion algorithm had an offset of 2cm and 3.5cm for each driving path in both static and dynamic environments, respectively. Reference [8] had an offset of approximately 3.6cm and 4.1cm for each driving path in both static and dynamic environments, while Reference [11] had an offset of approximately 3.9cm and 4.5cm for each driving path in both static and dynamic environments. The above results indicated that the fusion algorithm could still drive stably in various scenarios with moving obstacles and complex snowy scenes, while generating reasonable obstacle avoidance paths, ensuring the robustness of the positioning accuracy of wheeled robots.

## 5. Conclusion

Experimental results indicated that in the RI and SIF datasets, the OMFF-SLAM algorithm achieved the highest average precision, surpassing the BWFIN-SLAM algorithm and LiDARO-SLAM algorithm by 9.83% and 16.34%, and 3.04% and 4.67%, respectively. Moreover, compared to the state-of-the-art LiDARO-SLAM algorithm, the OMFF-SLAM algorithm demonstrated a reduction in processing time by 12.6ms. In slip detection experiments, the IMS-VSLAM algorithm accurately identified instances of slipping. In indoor datasets, the IMS-VSLAM algorithm exhibited an average precision of 85.4% and an average processing time of 64.4ms. In OD, the WCLD-IMS-VSLAM algorithm outperformed the WOCLD-IMS-VSLAM algorithm by 14.51% in average precision, with the IMS-VSLAM algorithm averaging a processing time of 91.63ms. In summary, the proposed research methods have facilitated wheeled robots in achieving high accuracy and real-time performance in complex scenarios, meeting the functional requirements of wheeled robots and demonstrating superior effectiveness in practical applications. However, there are still shortcomings in the research. The separation of multiple features and sensors into two steps in the proposed methods could be further improved to enhance the algorithm’s real-time capabilities. Future research could explore more advanced approaches to integrate multiple features and sensors into a unified algorithm.

## Supporting information

S1 Data set (DOCX)
