# Supplementary material for: Vision SLAM algorithm for wheeled robots integrating multiple sensors
Source: PLoS One. 2024 Mar 28;19(3):e0301189. doi: 10.1371/journal.pone.0301189 (PMC10977683; doi:10.1371/journal.pone.0301189)
Supplement: S1 Data set — (DOCX) [file pone.0301189.s001.docx]

Minimal Data Set Definition

**The data in Figure 9**

| Epoch | F1 | | | |
| --- | --- | --- | --- | --- |
|  | Improved model | Without dropout | SVM | KNN |
| 0 | 0.00 | 0.00 | 0.00 | 0.00 |
| 25 | 0.57 | 0.46 | 0.43 | 0.41 |
| 50 | 0.89 | 0.82 | 0.77 | 0.68 |
| 75 | 0.88 | 0.83 | 0.78 | 0.67 |
| 100 | 0.89 | 0.83 | 0.77 | 0.66 |
| 125 | 0.87 | 0.82 | 0.78 | 0.68 |
| 150 | 0.88 | 0.83 | 0.77 | 0.67 |
| 175 | 0.88 | 0.82 | 0.76 | 0.68 |
| Epoch | Recall | | | |
|  | Improved model | Without dropout | SVM | KNN |
| 0 | 0.00 | 0.00 | 0.00 | 0.00 |
| 25 | 0.71 | 0.58 | 0.56 | 0.37 |
| 50 | 0.82 | 0.81 | 0.67 | 0.52 |
| 75 | 0.83 | 0.79 | 0.66 | 0.53 |
| 100 | 0.82 | 0.78 | 0.67 | 0.53 |
| 125 | 0.83 | 0.78 | 0.67 | 0.53 |
| 150 | 0.82 | 0.77 | 0.66 | 0.52 |
| 175 | 0.82 | 0.78 | 0.67 | 0.52 |

**The data in Figure 10**

| Epoch | Response time | | | |
| --- | --- | --- | --- | --- |
|  | Improved model | Without dropout | SVM | KNN |
| 0 | 0.81 | 0.92 | 0.98 | 1.01 |
| 25 | 0.79 | 0.81 | 0.82 | 0.84 |
| 50 | 0.57 | 0.68 | 0.73 | 0.75 |
| 75 | 0.32 | 0.59 | 0.64 | 0.69 |
| 100 | 0.14 | 0.37 | 0.45 | 0.47 |
| 125 | 0.12 | 0.24 | 0.34 | 0.36 |
| 150 | 0.11 | 0.22 | 0.32 | 0.35 |
| 175 | 0.10 | 0.21 | 0.31 | 0.34 |

**The data in Figure 11**

| - | Post permission | Prerequisite permissions |
| --- | --- | --- |
| Improved model | 97.9 | 96.8 |
| Without dropout | 93.1 | 94.2 |
| SVM | 89.4 | 91.7 |
| KNN | 88.6 | 85.2 |

**The data in Figure 12**

| - | Large dataset test results-Accurate rate | | | |
| --- | --- | --- | --- | --- |
|  | Improved model | Without dropout | SVM | KNN |
| Description only-Post | 96.7 | 81.2 | 77.6 | 75.6 |
| Description only-Pre | 95.2 | 81.4 | 78.4 | 76.4 |
| Cvss_Mask-Post | 97.8 | 82.6 | 79.8 | 77.6 |
| Cvss_Mask-Pre | 96.4 | 84.3 | 79.6 | 76.4 |
| Level_Mask-Post | 96.4 | 87.5 | 78.4 | 78.6 |
| Level_Mask-Pre | 95.1 | 86.1 | 77.8 | 77.4 |
| Cpe_Mask-Post | 95.4 | 87.4 | 78.9 | 76.5 |
| Cpe_Mask-Pre | 96.5 | 86.2 | 80.2 | 77.2 |
| 3Score_Mask-Post | 95.5 | 86.1 | 81.7 | 78.6 |
| 3Score_Mask-Pre | 96.7 | 84.6 | 82.6 | 79.8 |
| Cwe_data_Mask-Post | 97.2 | 85.2 | 81.5 | 79.7 |
| Cwe_data_Mask-Pre | 97.9 | 85.4 | 81.1 | 78.6 |
| NoMask-Post | 97.1 | 86.4 | 82.6 | 81.6 |
| NoMask-Pre | 97.8 | 86.7 | 83.5 | 81.8 |
| - | Small dataset test results-Accurate rate | | | |
|  | Improved model | Without dropout | SVM | KNN |
| Description only-Post | 89.6 | 88.6 | 72.5 | 64.1 |
| Description only-Pre | 87.5 | 89.6 | 73.6 | 59.8 |
| Cvss_Mask-Post | 92.6 | 83.6 | 77.4 | 65.1 |
| Cvss_Mask-Pre | 91.4 | 84.1 | 78.6 | 66.2 |
| Level_Mask-Post | 92.5 | 84.5 | 78.9 | 67.8 |
| Level_Mask-Pre | 91.3 | 82.4 | 82.1 | 68.4 |
| Cpe_Mask-Post | 92.5 | 89.1 | 81.4 | 69.2 |
| Cpe_Mask-Pre | 91.2 | 87.2 | 84.6 | 70.1 |
| 3Score_Mask-Post | 91.5 | 89.5 | 85.7 | 71.5 |
| 3Score_Mask-Pre | 92.6 | 91.7 | 84.1 | 72.9 |
| Cwe_data_Mask-Post | 92.4 | 92.7 | 85.4 | 72.4 |
| Cwe_data_Mask-Pre | 93.6 | 93.5 | 84.3 | 73.4 |
| NoMask-Post | 97.5 | 90.6 | 86.2 | 78.4 |
| NoMask-Pre | 98.1 | 92.4 | 85.1 | 82.7 |

**The data in Figure 13**

| Epoch | Large dataset test results-Error rate | | | |
| --- | --- | --- | --- | --- |
|  | Improved model | Without dropout | SVM | KNN |
| 0 | 1.00 | 1.00 | 1.00 | 1.00 |
| 25 | 0.81 | 0.83 | 0.83 | 0.82 |
| 50 | 0.52 | 0.72 | 0.75 | 0.79 |
| 75 | 0.34 | 0.56 | 0.62 | 0.73 |
| 100 | 0.16 | 0.38 | 0.43 | 0.49 |
| 125 | 0.14 | 0.26 | 0.41 | 0.45 |
| 150 | 0.12 | 0.21 | 0.37 | 0.35 |
| 175 | 0.12 | 0.19 | 0.27 | 0.29 |
| Epoch | Small dataset test results-Error rate | | | |
|  | Improved model | Without dropout | SVM | KNN |
| 0 | 1.00 | 1.00 | 1.00 | 1.00 |
| 25 | 0.83 | 0.89 | 0.92 | 0.93 |
| 50 | 0.67 | 0.82 | 0.84 | 0.87 |
| 75 | 0.46 | 0.77 | 0.81 | 0.85 |
| 100 | 0.38 | 0.71 | 0.79 | 0.81 |
| 125 | 0.21 | 0.59 | 0.67 | 0.68 |
| 150 | 0.19 | 0.51 | 0.62 | 0.64 |
| 175 | 0.18 | 0.49 | 0.58 | 0.59 |

**The data in Figure 14**

| Time | Accuracy rate | | | |
| --- | --- | --- | --- | --- |
|  | Improved model | Without dropout | FF | KNN |
| 5 | 97.6 | 79.4 | 71.4 | 53.4 |
| 10 | 97.8 | 81.7 | 72.4 | 68.7 |
| 15 | 98.1 | 82.6 | 73.8 | 62.4 |
| 20 | 96.4 | 86.4 | 75.6 | 65.7 |
| 25 | 97.1 | 87.8 | 77.5 | 67.1 |
| 30 | 97.2 | 83.4 | 81.4 | 71.4 |
| 35 | 97.8 | 87.6 | 81.6 | 69.8 |
| 40 | 97.9 | 89.4 | 84.5 | 72.1 |
| 45 | 98.6 | 90.1 | 85.7 | 75.4 |
| Time | Error rate | | | |
|  | Improved model | Without dropout | FF | KNN |
| 5 | 13.5 | 48.6 | 75.4 | 85.7 |
| 10 | 14.6 | 39.4 | 57.6 | 76.4 |
| 15 | 9.8 | 31.5 | 65.4 | 52.6 |
| 20 | 7.6 | 27.6 | 61.2 | 62.5 |
| 25 | 4.8 | 25.4 | 59.7 | 68.4 |
| 30 | 4.1 | 23.1 | 51.2 | 68.9 |
| 35 | 3.4 | 17.6 | 41.6 | 52.7 |
| 40 | 2.7 | 14.8 | 31.6 | 41.5 |
| 45 | 2.1 | 15.7 | 25.7 | 42.7 |

**The data in Figure 15**

| Epoch | Proportion of detection | | | |
| --- | --- | --- | --- | --- |
|  | Improved model | Without dropout | FF | KNN |
| 0 | 0.00 | 0.00 | 0.00 | 0.00 |
| 25 | 0.72 | 0.47 | 0.57 | 0.42 |
| 50 | 0.96 | 0.86 | 0.77 | 0.58 |
| 75 | 0.95 | 0.85 | 0.77 | 0.67 |
| 100 | 0.97 | 0.86 | 0.76 | 0.66 |
| 125 | 0.96 | 0.86 | 0.78 | 0.68 |
| 150 | 0.97 | 0.86 | 0.76 | 0.67 |
| 175 | 0.97 | 0.87 | 0.77 | 0.68 |
| Epoch | Proportion of miss | | | |
|  | Improved model | Without dropout | FF | KNN |
| 0 | 0.80 | 0.80 | 0.80 | 0.80 |
| 25 | 0.46 | 0.51 | 0.47 | 0.56 |
| 50 | 0.04 | 0.19 | 0.27 | 0.37 |
| 75 | 0.04 | 0.11 | 0.17 | 0.32 |
| 100 | 0.03 | 0.10 | 0.16 | 0.31 |
| 125 | 0.04 | 0.11 | 0.17 | 0.32 |
| 150 | 0.04 | 0.11 | 0.17 | 0.31 |
| 175 | 0.03 | 0.11 | 0.16 | 0.31 |
